# Perinatal foodborne titanium dioxide exposure-mediated dysbiosis predisposes mice to develop colitis through life

**DOI:** 10.1186/s12989-023-00555-5

**Published:** 2023-11-23

**Authors:** Caroline Carlé, Delphine Boucher, Luisa Morelli, Camille Larue, Ekaterina Ovtchinnikova, Louise Battut, Kawthar Boumessid, Melvin Airaud, Muriel Quaranta-Nicaise, Jean-Luc Ravanat, Gilles Dietrich, Sandrine Menard, Gérard Eberl, Nicolas Barnich, Emmanuel Mas, Marie Carriere, Ziad Al Nabhani, Frédérick Barreau

**Affiliations:** 1 Institut de Recherche en Santé Digestive (IRSD), INSERM UMR-1220, Purpan Hospital, CS60039, University of Toulouse, INSERM, INRAE, ENVT, UPS, 31024 Toulouse Cedex 03, France; 2 M2iSH, Université Clermont Auvergne, UMR1071 INSERM, USC INRAE 1382, Clermont-Ferrand, France; 3 Department of Visceral Surgery and Medicine, Bern University Hospital, University of Bern, 3010 Bern, Switzerland; 4 Maurice Müller Laboratories, Department for Biomedical Research, University of Bern, 3008 Bern, Switzerland; 5 Laboratoire Ecologie Fonctionnelle et Environnement, Université de Toulouse, CNRS, Toulouse, France; 6 Univ. Grenoble-Alpes, CEA, CNRS, IRIG-SyMMES, CIBEST, Grenoble, France; 7 Institut Pasteur, Microenvironment and Immunity Unit, 75724 Paris, France; 8 INSERM U1224, Paris, France; 9 Gastroenterology, Hepatology, Nutrition, Diabetology and Hereditary Metabolic Diseases Unit, Hôpital des Enfants, CHU de Toulouse, 31300 Toulouse, France

**Keywords:** Perinatal period, Foodborne TiO_2_, Intestinal barrier function, Intestinal stem cells, Microbiota, Colitis

## Abstract

**Background:**

Perinatal exposure to titanium dioxide (TiO_2_), as a foodborne particle, may influence the intestinal barrier function and the susceptibility to develop inflammatory bowel diseases (IBD) later in life. Here, we investigate the impact of perinatal foodborne TiO_2_ exposure on the intestinal mucosal function and the susceptibility to develop IBD-associated colitis. Pregnant and lactating mother mice were exposed to TiO_2_ until pups weaning and the gut microbiota and intestinal barrier function of their offspring was assessed at day 30 post-birth (weaning) and at adult age (50 days). Epigenetic marks was studied by DNA methylation profile measuring the level of 5-methyl-2′-deoxycytosine (5-Me-dC) in DNA from colic epithelial cells. The susceptibility to develop IBD has been monitored using dextran-sulfate sodium (DSS)-induced colitis model. Germ-free mice were used to define whether microbial transfer influence the mucosal homeostasis and subsequent exacerbation of DSS-induced colitis.

**Results:**

In pregnant and lactating mice, foodborne TiO_2_ was able to translocate across the host barriers including gut, placenta and mammary gland to reach embryos and pups, respectively. This passage modified the chemical element composition of foetus, and spleen and liver of mothers and their offspring. We showed that perinatal exposure to TiO_2_ early in life alters the gut microbiota composition, increases the intestinal epithelial permeability and enhances the colonic cytokines and myosin light chain kinase expression. Moreover, perinatal exposure to TiO_2_ also modifies the abilities of intestinal stem cells to survive, grow and generate a functional epithelium. Maternal TiO_2_ exposure increases the susceptibility of offspring mice to develop severe DSS-induced colitis later in life. Finally, transfer of TiO_2_-induced microbiota dysbiosis to pregnant germ-free mice affects the homeostasis of the intestinal mucosal barrier early in life and confers an increased susceptibility to develop colitis in adult offspring.

**Conclusions:**

Our findings indicate that foodborne TiO_2_ consumption during the perinatal period has negative long-lasting consequences on the development of the intestinal mucosal barrier toward higher colitis susceptibility. This demonstrates to which extent environmental factors influence the microbial-host interplay and impact the long-term mucosal homeostasis.

**Supplementary Information:**

The online version contains supplementary material available at 10.1186/s12989-023-00555-5.

## Background

The gastrointestinal tract is a complex interface between the external environment and the immune system, establishing a dynamic barrier that enables the absorption of dietary nutrients and the exclusion of harmful compounds from the intestinal lumen, while permitting the sampling of luminal antigens as part of immune surveillance [1]. The ability to control uptake across the mucosa and protect from damage of harmful substances from the lumen is defined as the *intestinal barrier function (IBF)* [1]. The first line of defence is the commensal bacteria that produce anti-microbial substances and compete with pathogenic bacteria for nutrients [2]. The second line of defence is the mucus layer, which is a mechanical barrier against toxic substances and is rich in secreted IgA and antimicrobial peptides (AMP), thereby preventing the access of bacteria to the epithelium [3]. The third component of the barrier is composed of the monolayer of intestinal epithelial cells that further refrains toxic molecules and pathogens from entering the tissues. This constitutes the last defence before the mucosal immune system, which represents the ultimate barrier. Microbiota, intestinal epithelium and associated immune system works and regulate one another via tight relationships. The passage of nutrients and toxic substances from the lumen through the epithelium is achieved by both paracellular and transcellular permeabilities [1, 4].

Gut mucosal tissue homeostasis in adults results from the perinatal establishment of mucosally induced immune tolerance [5]. Perinatal defects in the induction of mucosal tolerance are associated with the development of numerous human diseases including allergies, autoimmune diseases or inflammatory bowel diseases (IBD) later in life [5]. Perinatal tolerance is induced by innate immune cells shaping adaptive immune response [6]. The intestinal epithelium controls this regulatory immune network through its barrier function, cell contact-mediated signals and the production of cytokines [5]. Perinatal exposure to cigarette smoke, dietary compounds or other environmental microorganisms plays a decisive role in maturation of the mucosal immune system [5, 7].

A significant number of human chronic diseases (inflammatory, metabolic …) is linked to a deficiency of the IBF and some of them, like IBD, exhibit alterations of the three IBF’s compartments [8, 9]. IBD is currently thought to be linked to environmental factors associated with the occidental way of life, which are currently unknown. Although cigarette smoking [10] or carbohydrate intake [11] are associated with IBD, other environmental factors such as ingestion of food additives (FA) and emulsifiers may present potential health risks by altering the IBF and then favouring IBD [12–14].

Among FA, titanium dioxide (TiO_2_) is commonly-used as a white pigment (E171), and it is frequently added to food like candies, dairy products, and beverages, to mainly improve colour and consistency [15]. Foodborne TiO_2_ particles range from nanoparticles to micro-particles, and some studies have shown that this FA contains at least 36% of nanoscale TiO_2_ particles [16, 17]. E171 is among the most commonly used mineral particle-based FA in consumer products [16, 18–20]. As largely described, acute or chronic exposure to TiO_2_ in adult rodents alters the elements of the IBF including microbiota, epithelium and gut immune system [14, 21, 22]. Nevertheless, the underlying mechanisms are poorly understood, and whether these changes are reversible or not when the exposure stops is currently unknown.

In addition, E171 is able to translocate into the internal body [21, 23, 24]. Finally, exposure of adult mice to TiO_2_ is described not only to induce severe gut inflammation following DSS administration [25], but also the patients with active IBD present higher levels of TiO_2_ in their systemic circulation, compared to healthy people [25].

Up to now, the majority of TiO_2_ FA intestinal toxicity studies have been performed on models reproducing the consequences of adult exposure, and few data are available concerning exposure to TiO_2_ early in life. One recent study reported that young rats are more susceptible to oral exposure to TiO_2_ particles and showed more adverse reactions than adult animals [26]. This lack of data is worrying because the higher levels of TiO_2_ are found in products that children eat in large quantities (sweets, candies etc.), leading to the highest exposure to foodborne TiO_2_ of children [16, 27]. A recent study reported that although the risk of high exposure to foodborne TiO_2_ particles in children has arisen the attention of the academic community, only a few studies have focused on this specific age group [28] while no study is available on the likely impact during the pregnancy and lactating periods.

In this study, we hypothesized that perinatal exposure to TiO_2_ could alter the elements of the IBF, then favouring the development of intestinal inflammation throughout life. To evaluate this hypothesis, we exposed pregnant female C57BL/6 mice to 9 mg E171/kg b.w./day via their drinking water, from the beginning of gestation until 4 weeks post-delivery. Then, their offspring were exposed first via drinking their mother’s milk, mothers being still exposed to 9 mg E171/kg b.w./day via their drinking water. After weaning, pups were directly via drinking water, also containing 9 mg E171/kg b.w./day, until postnatal day 50. This exposure concentration is in the lower range of the estimated daily exposure of human adults, which ranges between 5.5 and 10.4 mg/kg b.w./day according to EFSA’s estimations [29]. When considering the guidances on dose conversion between human and animal exposure, such as the Nair and Jacob practice guide or FDA’s guidelines, we previously estimated that doses up to 50–60 mg/kg b.w./day in mice would be realistic [14] confirming that the dose used in the present study can be considered as a low exposure dose. This experimental strategy led us to evidence that TiO_2_ was able to translocate into embryos and pups, to alter their composition in chemical elements, as well as the development and the homeostasis of the intestine. Moreover, we deciphered the mechanisms by which perinatal exposure to TiO_2_ altered the homeostasis of the IBF and the enhanced susceptibility to develop colitis throughout life and highlight the role of microbiota.

## Results

### Translocation of TiO_2_ from mother to offspring mice

**Fig. 1:**
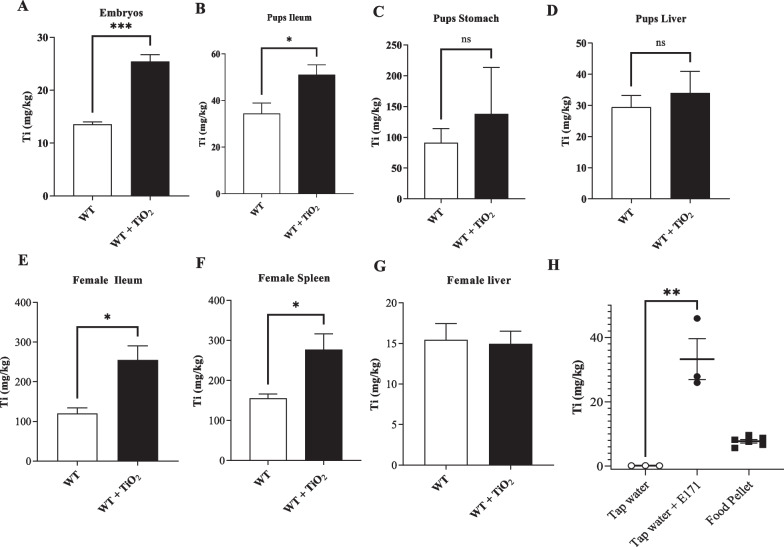
Abilities of foodborne TiO_2_ to translocate across the human barriers. **A**–**G** Wild type female mice have been exposed to TiO_2_ (9 mg/Kg of BW/Day) during the perinatal period including gestational and lactating periods. Then pregnant and lactating females exposed to TiO_2_ or not, have been sacrificed and the concentrations of Ti in embryos (**A**; gestational day 20), pup’s organs (**B**–**D**; postnatal day 12) and in female organs (**E**–**G**; end of the weaning day 30), have been monitored by ICP-MS. **H** The concentrations of titanium have been determined by ICP-MS in regular food pellet and in tap water before and after the addition of TiO_2_ foodborne to reach the exposure dose: 9 mg of TiO_2_/kg of body weight/day. Data are expressed as mean ± SEM and were analysed by Student’s t-test. *p < 0.05, **p < 0.01 and ***p < 0.001 versus control group. At least n = 3 per group

To determine whether TiO_2_ can be transferred from mother to their offspring, pregnant female mice were exposed to TiO_2_ (9 mg/kg of body weight/day) in drinking water until weaning and the Ti concentration was determined by ICP-MS. Compared to control mice exposed to water only, we observed a significant increase of Ti concentration in foetus tissues measured at embryonic day 20 (Fig. 1A) confirming the translocation of TiO_2_ from the mother to the embryos. In the ileum of pups, as measured at postnatal day 12, increased Ti contents were also measured as compared to non-exposed animals (Fig. 1B) but no significant increase was observed in their stomach or liver (Fig. 1C, D). This suggests that TiO2 got adsorbed on the ileum’s surface and may potentially translocate through the intestine of pups, although this would need to be confirmed by Ti quantification in the pup’s bloodstream. However, it is difficult to sample due to the age and size of puppies. Mother mice at day 21 post-delivery (weaning) had also a significant increase of Ti concentration in the ileum and spleen tissues but not in the liver compared to untreated mice (Fig. 1E–G). Although the quantities of Ti in tap water were around 0.5 mg/kg (Fig. 1H), the control mice which were not exposed to TiO_2_ had elevated Ti concentration probably due to the presence of Ti in food pellet (Fig. 1H). Taken together, these data showed that TiO_2_ can be found in offspring after their mother's exposure.

To investigate the impact of TiO_2_ exposure on the concentrations of different chemical elements in organs, we compared the frequency of a large number of chemical elements in TiO_2_-exposed mother mice to unexposed control. We showed that exposure to foodborne TiO_2_ increased significantly the concentrations of Boron (B), Sodium (Na), Magnesium (Mg) and Calcium (Ca) in the spleen but TiO_2_ exposure did not modify the composition of chemical elements in liver (Additional file 1: Fig. S1A). In addition, maternal exposure to foodborne TiO_2_ influences the composition of chemical elements in both embryos and pups (Additional file 1: Fig. S1B, C). Indeed, while the concentrations of B, Aluminium (Al), Silicon (Si), Potassium (K), Chromium (Cr), Manganese (Mn), Cobalt (Co), Nickel (Ni), Copper (Cu), Zinc (Zn), Selenium (Se) and Lead (Pb) were increased, the concentrations of Mg, Phosphorus (P) and Ca were reduced in embryos exposed to TiO_2_ (Additional file 1: Fig. S1B). Perinatal exposure to foodborne TiO_2_ is associated with increased concentrations of B, Ca, Cr, Mn, Iron (Fe), Nickel (Ni) and Cu and a reduced concentration of P in pup’s liver compared to control (Additional file 1: Fig. S1C). While Ti reached embryos and affected the chemical element composition, the perinatal exposure to foodborne TiO_2_ had no impact on the foetus survival as well as on the male/female ratio (Additional file 2: Fig. S2).

### Maternal exposure to TiO_2_ leads to microbiota dysbiosis in young and adult offspring

**Fig. 2:**
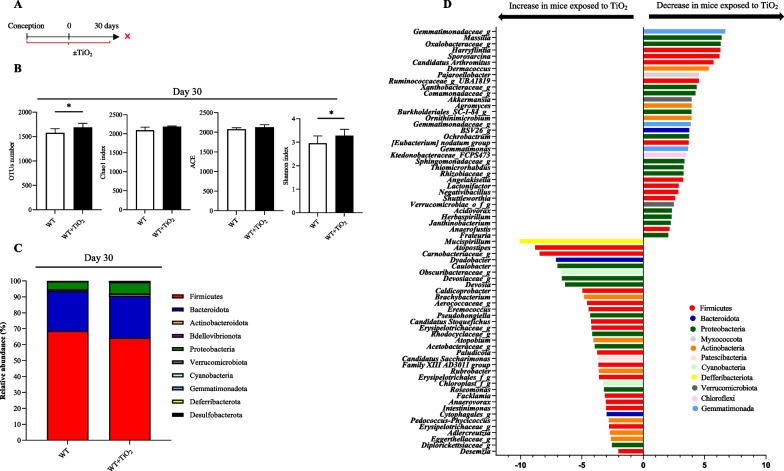
Impact of perinatal exposure to foodborne TiO_2_ on colonic microbiota at day 30. **A**–**D** Wild type female mice have been exposed to TiO_2_ (9 mg/Kg of BW/Day) during the perinatal period including gestational and lactating periods. Then at 30 days after birth, pups have been sacrificed and the structure of the colonic mucosa-associated microbiota has been monitored by 16S rRNA gene sequencing (**B**–**D**). **B** Alpha diversity of colonic mucosal microbiota from exposed or non-exposed mice to foodborne TiO_2_ at day 30 after birth. **C** Composition of colonic microbiota at phyla level (**C**) and Fold changes 2 for bacterial genera significantly perturbed (**D**) from exposed or non-exposed mice to foodborne TiO_2_ at day 30 after birth. Data are expressed as median ± SEM and were analysed by Mann and Whitney test. *p < 0.05 versus control group. At least n = 8 per group

To determine the consequences of exposure of pregnant dams to TiO_2_ on the microbial communities of their offspring, the colonic mucosa was subjected to 16S rRNA gene sequencing using Illumina NextSeq500. We chose to investigate the bacterial communities associated with the colonic mucosa due to their proximity to underlying epithelium and their potential role in the exchange of nutrients and the induction of the host innate immune system. Comparing the colonic-associated microbiota composition in the offspring of mother mice exposed to TiO_2_ to untreated control mice (Fig. 2A; Additional file 3: Fig. S3A), we showed that both bacterial richness and diversity were significantly higher at day 30 but not at the day 50 in colonic mucosa as measured by OTUs number and Shannon index (Fig. 2B; Additional file 3: Fig. S3B). Regardless of the sample considered, members belonging to Firmicutes, followed by Bacteroidota, dominated the bacterial community composition at the phylum level (Fig. 2C; Additional file 3: Fig. S3C). If no significant impact of perinatal exposure of TiO_2_ have been observed for this taxonomic ranking at days 30 and 50, important alterations have been observed at genus level for both sampling dates (Fig. 2D; Additional file 3: Fig. S3D). At day 30 the most increased taxa in mice exposed to TiO_2_ belong mainly to Firmicutes (with namely *Atopostipes*), Pseudomonadota (i.e. *Caulobacter, Roseomonas*), Actinobacteriota but also to Bacteriodota and Defferibacteriota (*Muscispirillum*), while the most reduced taxa belong to Firmicutes (*Candidatus arthromitus)*, Pseudomonadota, Actinobacteria, Gemmatimonada and Verrucomicrobiota (*Akkermansia*) (Fig. 2D). At day 50, TiO_2_ exposure in mice led to a significant increase of bacteria affiliated to the phyla of Firmicutes, Pseudomonadota (*Caulobacter, Brevundimonas*) and Bacteroidota and a decrease of taxa belonging mainly to Firmicutes (Additional file 3: Fig. S3D).

### Perinatal exposure to TiO_2_ alters the functionality and the renewal of the colonic epithelium in young and adult mice

**Fig. 3:**
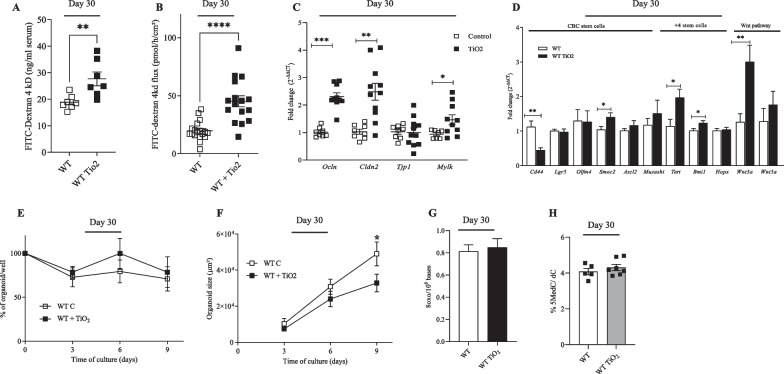
Impact of perinatal exposure to foodborne TiO_2_ on colonic epithelium at day 30. **A**–**D** Wild type female mice have been exposed to TiO_2_ (9 mg/Kg of BW/Day) during the perinatal period including gestational and lactating periods. Then at days 30 after birth (weaning), pups have been sacrificed and several parameters including permeability (**A** and **B**), mRNA expression (**C** and **D**), abilities of intestinal stem cells to survive (**E**) and proliferate (**F**), oxidative stress (**G**) and DNA methylation (**H**) were assessed. **A** In vivo permeability was determined by measuring the level of plasmatic FITC-dextran 4 kDa, 3 h following oral administration. **B** Colonic permeability was monitored by measuring the flux of FITC-dextran 4 kDa across colonic biopsies mounted in Ussing chamber for 1 h. **C** mRNA expression of Occludin (*Ocl*), Claudin 2 (*Cldn 2*) Tight junction protein 1 (*Tpj1*) and myosin light chain kinase (*Mlck*) was studied at days 30 and 50 after birth. **D** Colonic mRNA expression of CD44, Leucine-rich repeat-containing G-protein coupled receptor 5 (*Lgr5*), Olfactome-din 4 (*Olfm4*), SPARC-related modular calcium-binding protein 2 (*Smoc2*), Achaete-scute complex homolog 2 (*Ascl2*), Musashi RNA-binding protein 1 (*Musashi*), Telomerase reverse transcriptase (*Tert*) and B lymphoma Mo-MLV insertion region 1 homolog (*Bmi1*), homeodomain-only protein homeobox (*Hopx*), canonical (*Wnt3a*) and non-canonical (*Wnt5a*). **E** The organoid survival has been monitored by measuring the percentage of viable organoids according to the time culture. **F** The organoid growth has been studied by measuring the organoid surface according to the time culture. **G** Oxidative stress has been monitored into the epithelial cells from the colonic based crypt of mice perinatally exposed or not to TiO_2_. **H** The quantity of cytosine methylated of DNA from the epithelial cells from the colonic based crypt of mice perinatally exposed or not to TiO_2_. Data are expressed as mean ± SEM and were analysed by Student’s t-test. *p < 0.05; **p < 0.01; ***p < 0.001 and ****p < 0.0001 vs control group. At least n = 8 per group

Since gut microbiota is described to modulate the intestinal epithelium homeostasis [30, 31], we investigated if perinatal exposure to foodborne TiO_2_ also alters the integrity of the gut epithelium (Fig. 3; Additional file 4: Fig. S4). At both days 30 and 50 after birth, perinatal exposure to TiO_2_ increased significantly the flux of Dextran-FITC 4kD across the digestive mucosa, in vivo (Fig. 3A; Additional file 4: Fig. S4A). This was confirmed in Ussing chamber experiments, where perinatal exposure to TiO_2_ was also shown to increase the flux of Dextran-FITC 4kD across the colonic epithelium from mice at days 30 and 50 after birth (Fig. 3B; Additional file 4: Fig. S4B). In addition, the expression of myosin light chain kinase (*Mylk*), a master regulator of the tight junction opening [32], was increased by perinatal exposure to TiO_2_ at days 30 and 50 after birth (Fig. 3C; Additional file 4: Fig. S4C). While perinatal exposure to TiO_2_ increased the mRNA level of Claudin 2 (*Cld2*) in mice colon at days 30, its expression was not changed at days 50 (Fig. 3C; Additional file 4: Fig. S4C). In contrast to *Cld2*, mRNA level of Tight junction protein 1 (*Tpj1*) was unaffected at days 30 while its expression was increased at days 50 after birth (Fig. 3C; Additional file 4: Fig. S4C). Finally, perinatal exposure to TiO_2_ increased the mRNA level of colonic Occludin (*Ocln*) at days 30 while its expression was reduced at days 50 after birth (Fig. 3C; Additional file 4: Fig. S4C).

At days 30 after birth, perinatal exposure increased the mRNA levels of *Muc2*, *Muc3*, *Muc4* and *Tff3* while it decreased the faecal levels of lysozyme (Additional file 5: Fig. S5A–C). At days 50 after birth, TiO_2_ exposure only increased the level of *Muc2* (Additional file 5: Fig. S5A).

**Fig. 4:**
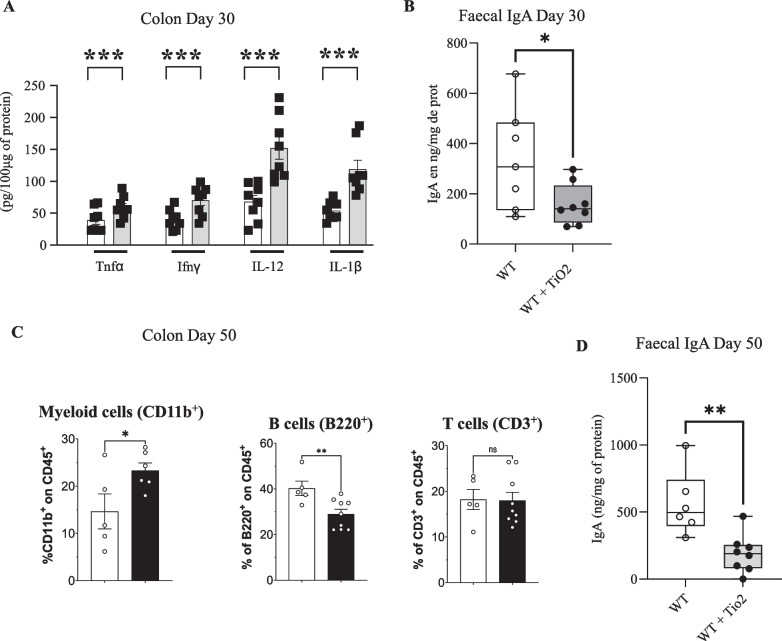
Impact of perinatal exposure to TiO_2_ foodborne on intestinal immune system. **A**–**D** Wild type female mice have been exposed to TiO_2_ (9 mg/kg of BW/Day) during the perinatal period including gestational and lactating periods. **A** and **B** Then, at day 30 after birth, pups have been sacrificed and colonic protein expression of cytokines (**A**) and faecal levels of IgA (**B**) have been monitored. **C** and **D** At day 50 after birth, immune cells population of the colon (**C**) and faecal levels of IgA (**D**) have been measured. Data are expressed as mean ± SEM and were analysed by Student’s t-test. *p < 0.05 and **p < 0.01 versus control group. At least n = 8 per group

Since perinatal exposure to TiO_2_ altered the functionality of the colonic epithelium, we then monitored its effects on the intestinal epithelial stem cells (IESC) homeostasis (Fig. 4D–F; Additional file 5: Fig. S4D–F). Following extraction of the base crypt containing IESC, we determined the mRNA levels of some markers of immaturity, crypt base columnar (CBC) stem cells, + 4 stem cells and Wnt pathway in the mice colon at days 30 and 50 after birth (Fig. 3D; Additional file 4: Fig. S4). At post-natal day 30, mice exposed to TiO_2_ showed decreased mRNA level of CD44, a marker of immaturity, while it increased the mRNA level of SPARC-related modular calcium-binding protein 2 (*Smoc2*), a marker of CBC stem cells, telomerase reverse transcriptase (*Tert*) and B lymphoma Mo-MLV insertion region 1 homolog (*Bmi1*), two markers of + 4 stem cells, as well as the marker of canonical wnt pathway (wnt3a) (Fig. 3D). At day 50, mice exposed to TiO_2_ had an increased mRNA levels of colonic CD44, Leucine-rich repeat-containing G-protein coupled receptor 5 (*Lgr5*), Achaete-scute complex homolog 2 (*Ascl2*) and Musashi RNA-binding protein 1 (*Musashi*), three markers of CBC, Telomerase reverse transcriptase (*Tert*) and Homeodomain-only protein X (*Hopx*), two markers of + 4 stem cells and the marker of non-canonical wnt pathway (wnt5, involved in inflammatory pathway) (Additional file 4: Fig. S4D) but not Olfactomedin-4 *(Olfm4*) (Additional file 4: Fig. S4D).

Next, we investigated the ability of IESC to survive and grow to generate organoids by cultivating for 9 days the base of the crypts obtained from TiO_2_-exposed mice or their control at 30 or 50 days of age (Fig. 3E, F; Additional file 4: Fig S4E, F). We observed a significant reduction of organoid growth at day 9 post-organoid culture obtained from TiO_2_-exposed mice compared to control at day 30 (Fig. 3F) but the survival of colonic organoids was similar between both TiO_2_-treated and untreated group (Fig. 3E). At day 50 after birth, in line with the increased mRNA levels of some stem cells and wnt pathway markers, mice exposure to TiO_2_ had a reduced colonic organoid survival while it enhanced the growth of organoids (Additional file 4: Fig. S4E, F).

Finally, since oxidative stress and/or DNA methylation are well known to regulate gene expression, we monitored the impact of exposure to TiO_2_ on the oxidative balance as well as DNA methylation of the colonic epithelium (Fig. 3G, H; Additional file 4: Fig. S4G). In this objective, we used 8-oxo-dGuo as a biomarker of DNA oxidation, this lesion being also considered as a marker of oxidative stress [33] and being quantifiable with a high sensitivity using methods such as HPLC-tandem mass spectrometry [34]. As a DNA methylation biomarker, we quantified 5-methyl-2′-deoxycitidine, i.e., 5-Me-dC, as it is the predominant methylation site in mammalian genomes and it shows the highest biological significance as it modulates the binding of transcription factors to DNA [29, 35]. Again, we used HPLC-tandem mass spectrometry to quantify this DNA base [36, 37]. As evidenced in Fig. 3G and in Additional file 4: Fig. S4G, perinatal exposure to TiO_2_ did not trigger DNA oxidation, which suggests that it did not trigger oxidative stress, as shown by unchanged levels of 8-oxo-dGuo in epithelial cells at days 30 and 50 after birth. However, perinatal exposure to TiO_2_ increased DNA methylation, as evidenced by elevated 5-Me-dC in colonic epithelial cells at day 50 but not at day 30 after birth (Fig. 3H; Additional file 4: Fig. S4H).

### Perinatal exposure to TiO_2_ alters the homeostasis of the gut immune system

In addition to colonic microbiota, mucus layer and intestinal epithelium, we investigated the significant consequences of perinatal exposure to TiO_2_ on the colonic immune system homeostasis in young (Day 30) and adult mice (Day 50). As illustrated in Additional file 6: Fig. S6A, B, perinatal exposure to foodborne TiO_2_ increased the mRNA levels of *Il1b*, *Il6*, *Il12b*, *Il22* and *Tnfa* in Peyer’s patches and ileum at day 30 after birth. This profile was further amplified at day 50 after birth, where stronger increases of IL—*Il1b*, *Il6*, *Il10*, *Il12b*, *Il22*, *Tnfa* and *Ifng* mRNA expression were observed (Additional file 6: Fig. S6A, B). In the colon, at day 30 after birth only reduced expression of *Il10* and *Il23* were observed, as well as increased expression of *Il22* (Additional file 6: Fig. S6C). In contrast to those observed in colon of young mice, perinatal exposure to TiO_2_ did not affect the mRNA level of *Il23* at day 50 while it increased the expression of *Il1b*, *Il6*, *Il10*, *Il22*,* Tnfa* and* Ifng* (Additional file 6: Fig. S6C). However, at protein level, perinatal exposure to TiO2 increased the colonic cytokines expression of Tnfα, Ifnγ, IL-12 and IL-14 at day 30 (Fig. 4A).

Regarding colonic immune cell populations, flow cytometry experiments on the lamina propria from colon of mice (day 50) evidenced that perinatal exposure to TiO_2_ increased the percentage of myeloid cells (CD11b^+^), reduced the percentage of B cells (B220^+^) and did not change the percentage of T-cells (CD3^+^) (Fig. 4C). Finally, the reduced percentage of B cells in the lamina propria was associated with reduced faecal levels of IgA, but not IgG at both days 30 and 50 after birth (Fig. 4B–D; Additional file 5: Fig. S5D).

### Microbiota dysbiosis induced by perinatal exposure to TiO_2_ alters the homeostasis of colonic mucosa

**Fig. 5:**
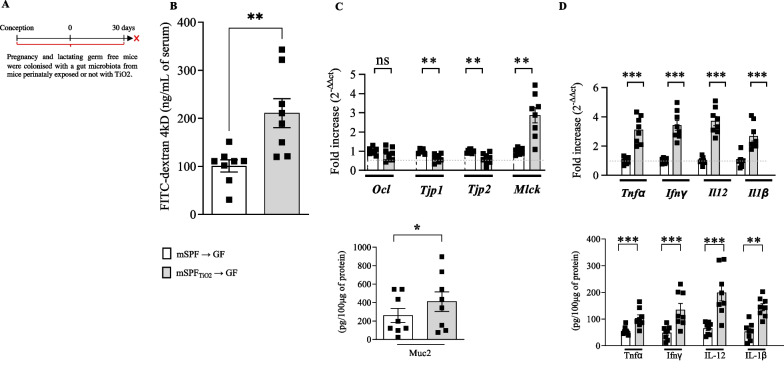
Impact of gut microbiota dysbiosis induced by perinatal exposure to foodborne TiO_2_ on the intestinal barrier function of offspring. **A**–**D** Germ free female mice have been exposed to gut microbiota dysbiosis induced by perinatal foodborne until the weaning i.e. postnatal days 30. **B** Then, i*n vivo* permeability was determined by measuring the level of plasmatic FITC-dextran 4 kDa, 3 h following oral administration. **C**, **D** On scrapped colonic epithelium, mRNA expression of *Il1b*, *Il12*, *Tnfa*, *Ifng,* Occludin (*Ocl*), Tight junction protein 1 (*Tpj1*) and Tight junction protein 2 (*Tpj2*) and myosin light chain kinase (*Mlck*) have been monitored at postnatal days 30. Data are expressed as mean ± SEM and were analysed by Student’s t-test. **p < 0.01 and ***p < 0.001 versus control group. At least n = 5 per group

Since gut microbiota dysbiosis has been shown to alter the gut homeostasis [7, 30, 38], we assessed if the observed modified functionality of the colonic mucosa was mediated by TiO_2_-induced microbial dysbiosis. Therefore, germ-free pregnant mice were colonized with faecal microbiota from mice exposed to TiO_2_ or with microbiota from untreated control (Fig. 5A). Six weeks after microbiota transfer, permeability and mRNA levels of *Occludin*, *Tpj1*, *Tpj2* and *Mylk* as well as *Il1b*, *Il12*, *Tnfa* and *Ifng* were assessed (Fig. 5B–D). As illustrated in Fig. 5B, the transfer of TiO_2_-triggered microbiota dysbiosis to healthy germ-free mice led to significantly increased paracellular intestinal permeability (Fig. 5B), increased mRNA level of *Mylk*, and reduced mRNA level of *Tjp1* and Tjp2 (Fig. 5C) in offspring at day 30. In addition, these mice exhibited a pro-inflammatory status, as evidenced by an increased mRNA levels of *Il1b*, *Il12*, *Tnfa* and *Ifng* compared to mice having received control microbiota (Fig. 5D). Altogether, these data demonstrate that the effect of TiO_2_ on intestinal function is mediated by a microbiota-dependent mechanism.

### Perinatal exposure to TiO_2_ increases the colitis susceptibility throughout life

**Fig. 6:**
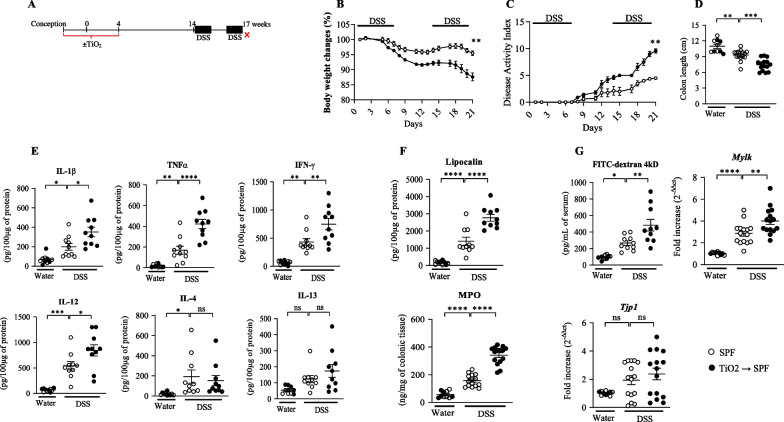
Impact of perinatal exposure to foodborne TiO_2_ on susceptibility to develop colitis later in life. **A**–**G** Wild type female mice have been exposed to TiO_2_ (9 mg/Kg of BW/Day) during the perinatal period including gestational and lactating periods (**A**). At 14 weeks of age, colitis has been orally induced by introducing Dextran Sulfate Sodium (DSS) into drinking water at 2% for 7 days followed by 7 days of regular water then 7 days of DSS (**A**). **B** and **C**, During the experimental DSS procedure (21 days), body weight and the disease activity index (DAI) have been monitored daily. **D**–**G** Then, at the end of the DSS procedure, mice have been sacrificed and colonic length (**D**) and, cytokine expression (**E**) have been monitored. **F** and **G** Finally, lipocalin, MPO, permeability and mRNA expression of *Mlck* and *Tpj1* have also been studied. Data are expressed as mean ± SEM and were analysed by Student’s t-test. *p < 0.05; **p < 0.01; ***p < 0.001 and ****p < 0.0001 versus control group. At least n = 5 per group

Since perinatal exposure to TiO_2_ altered the homeostasis of the IBF early in life at weaning, we investigated whether such alteration may persist in adulthood and their influence on colitis development (Fig. 6)—long lasting imprinting of perinatal exposure. We observed that alteration of homeostasis of the colonic mucosa related to early life exposure to TiO_2_ did not persist until adult 17 weeks of age as monitored for permeability, cytokine and other inflammatory markers i. e. in the group unchallenged for DSS mice exposed to TiO2 superpose with mice unexposed (Fig. 6; Additional file 7: Fig. S7). However, as illustrated in Fig. 6B–G, perinatal exposure to TiO_2_ enhanced significantly the loss of body weight and the DAI induced by DSS. Perinatal exposure to TiO_2_ also exacerbated the colitis, as evidenced by a reduced colon length associated with increased colonic mRNA expression and protein levels of IL-1β, IL-4, IL-12, IL-13, IFNγ and TNF-α (Additional file 6: Fig. S6A and Additional file 7: Fig. S7E). Moreover, perinatal exposure to TiO_2_ increased the faecal inflammatory markers such as lipocalin and MPO, which were induced by DSS (Fig. 6F). Perinatal exposure to TiO_2_ also aggravated significantly the alterations of intestinal permeability, as evidenced by an increased Dextran-FITC flux, mRNA expression of MLCK and a reduced mRNA level of Tjp1 (Fig. 6G).

### Microbiota dysbiosis induced by perinatal exposure to TiO_2_ increases the colitis susceptibility later in life

**Fig. 7:**
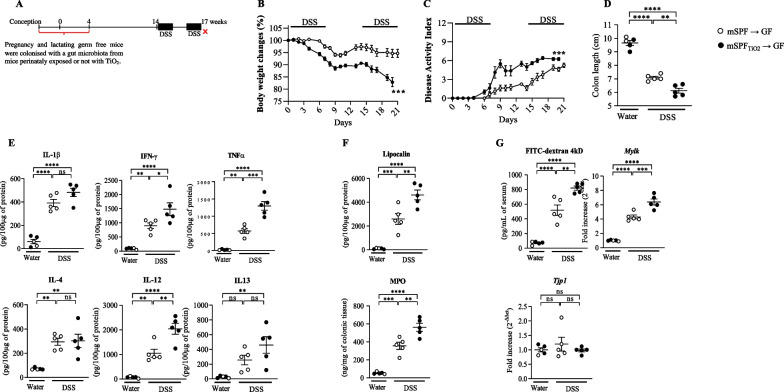
Impact of gut microbiota dysbiosis induced by perinatal exposure to TiO_2_ on the susceptibility to develop colitis later in life. **A**–**G** Germ free mice female have been exposed to gut microbiota dysbiosis induced by perinatal foodborne until the weaning i.e. postnatal day 30 (**A**). At 14 weeks of age, colitis has been orally induced by introducing Dextran Sulfate Sodium (DSS) into drinking water at 2% for 7 days followed by 7 days of regular water then 7 days of DSS (**A**). **B** and **C** During the experimental DSS procedure (21 days), body weight and the disease activity index (DAI) have been monitored daily. **D**–**G** Then, at the end of the DSS procedure, mice have been sacrificed and colonic length (**D**), cytokines expression (**E**) have been monitored. **F** and **G** Finally, lipocalin, MPO, permeability and mRNA expression of *Mlck* and *Tpj1* have also been studied. Data are expressed as mean ± SEM and were analysed by Student’s t-test. *p < 0.05; **p < 0.01; ***p < 0.001 and ****p < 0.0001 versus control group. At least n = 5 per group

Pregnant and lactating germ-free mice were colonized with gut microbiota from mice perinatally exposed to foodborne TiO_2_, which showed dysbiosis, as reported in Fig. 7 A. At postnatal day 30, pups from pregnant and lactating germ-free dams, colonized by faecal microbiota from mice exposed or not to foodborne TiO_2_, exhibited alterations of the intestinal barrier function including permeability and cytokine expression (Fig. 5). In contrast, at the 17th week of life, there was no longer any significant difference in terms of permeability, cytokine or other inflammatory markers i. e. in the group unchallenged for DSS mice exposed to TiO2 superpose with mice unexposed (Fig. 7E–G). Colitis was monitored by measuring body weight and DAI every day. As illustrated in Fig. 7B, C, the loss of body weight and DAI induced by DSS was enhanced in animals having received the dysbiotic microbiota of mice perinatally exposed to TiO_2_. The colitis was exacerbated in these animals, as evidenced by a reduced colon length associated with increased colonic mRNA expression and protein levels of IL-1β, IL-4, IL-12, IL-13, IFNγ and TNF-α (Additional file 7: Fig. S7E and file 8: Fig. S8). Moreover, it increased the expression of lipocalin and MPO faecal inflammatory markers (Fig. 7F). Finally, it aggravated the alterations of intestinal permeability, as evidenced by an increased 4kDa Dextran-FITC flux, mRNA expression of *Mlck* and a reduced mRNA expression of *Tjp1* (Fig. 7G).

## Discussion

We and others have shown that acute or chronic exposure to elevated doses of foodborne TiO_2_ in adult period can alter the IBF and/or aggravate the colitis [14, 18, 21, 22, 25, 39, 40]. Here, we report that in pregnant and lactating mice, low and realistic dose of foodborne TiO_2_ was able to translocate across the host barriers including gut, placenta and mammary gland to reach embryos and pups, respectively. As developed in the Material and Methods section, the dose used in this study corresponds to a daily ingestion of 5.5 mg/kg b.w., and the estimated human exposure ranges from 5.5 to 10.4 mg TiO2/kg b.w. per day [41]. In addition to this passage across host barrier, TiO_2_ exposure alters the composition of chemical elements in both embryos and pup liver. The consequences of this TiO_2_ perinatal passage are an altered IBF, associated with enhanced susceptibility to develop colitis lifelong. Moreover, our data evidence that microbiota dysbiosis induced by perinatal exposure to TiO_2_ is sufficient to alter the IBF and to increase the susceptibility to develop colitis lifelong. These results evidence that environmental factors such as foodborne TiO_2,_ by interacting with the microbiota, are able to alter the other components of the IBF to promote intestinal inflammation.

Despite the abilities of foodborne TiO_2_ to translocate across the intestinal epithelium, their fate in the internal body is unclear. According to the dose and the type of TiO_2_ particles, accumulations in spleen, liver and kidney have been reported [14]. However, concerning their potential abilities to translocate across the placenta and the mammary gland to reach embryos and pups, respectively, this was documented by only one recent study using an ex vivo placenta perfusion model [42]. In this study, authors evidenced that foodborne TiO_2_ particles were able to cross the cotyledon of human placenta while no data are available concerning their potential in vivo passage [42]. Moreover, the presence of Ti in the meconium does not indicate if its passage underwent during gestation and/or the beginning of suckling. In agreement with this study, our data evidence that foodborne TiO_2_ is able to translocate across the intestinal mucosa to reach the internal medium (spleen) of pregnant females and then the embryos by crossing the placenta. Moreover, as foodborne TiO_2_ particles have been monitored in the luminal content of pup’s small intestine fed by female exposed to TiO_2_, this suggests the ability of TiO_2_ to translocate from the luminal content of the mother gut to the breastmilk, then reaching pups. Whether TiO_2_ can translocate through the pup’s intestine cannot be proved with the current results, since the presence of Ti in the intestinal lumen does not necessarily mean that it would translocate. Ti should remain adherent to the pup’s intestinal epithelium and/or mucus layer without translocating towards the internal tissues. In addition to the abilities of foodborne TiO_2_ to reach embryos and pups, our data also demonstrate their deleterious impacts on the composition of chemical elements in both embryos and pup’s organ. The most affected elements are Boron, Aluminium, Silicon, Chromium, Cobalt, Nickel, Lead and Calcium which are reduced in embryos. To our knowledge no study has previously reported such impact of foodborne TiO_2_ on the composition of the chemical elements in embryos and in pup’s organ as well as their potential deleterious impact on the embryos and pup’s health.

An important literature has reported the deleterious impact of acute or chronic exposure to elevated doses of TiO_2_ on the intestinal mucosa from adult rodent [14]. Thus, altered faecal microbiota, colonic permeability and expression of some cytokines and chemokines have been reported [14]. In contrast to adult, only one study has reported that young rats were more susceptible to oral exposure to TiO_2_ particles and showed more adverse reactions than adult animals [26]. Young (3 week-old) but not old rats (8 week-old) exposed to TiO_2_ for 30 days exhibited liver oedema, heart injuries and mast cell activation in stomach [26]. In agreement with this study, our data evidence that perinatal exposure strongly alter all the elements of the intestinal barrier function i.e. mucosal microbiota, mucus layer, epithelium and immune system. Also, the perturbation of gut microbial structure with increase of potential pathobionts and decrease of benefit bacteria can lead to increased susceptibility to immunopathologies later in life. Among the most important alterations of the mucosal microbiota was the increase of *Muscispirillum* in mice exposed to TiO_2_ at day 30. This bacteria, which resides in the intestinal mucus layer harbors some virulence traits (type VI secretion system and putative effector proteins) [43], which can trigger IBD-like disease in the presence of impaired clearance of the bacterium by innate immunity [44]. In the same way, *Atopobium*, which has been shown to control the central hub of H_2_S producers and to cause colitis in a susceptible mouse model [45], are also increased by TiO_2_ exposure. In a different manner, *Candidatus arthromitus*, also known as segmented filamentous bacteria (SFB), is depleted by the exposure to TiO_2_ at day 30. SFB is a commensal bacterium necessary for inducing the postnatal maturation of gut immune inducing IgA response [46]. Its decrease could participate in the altered perinatal development of the immune system leading to pathological imprinting and increased susceptibility to colitis, and cancer later in life [7]. By transferring this microbiota dysbiosis into germ free mice, we have thus evidenced the negative impact of the microbiota dysbiosis on the intestinal homeostasis (increased permeability and cytokine expression) and increased susceptibility to develop colitis. The deleterious impact of this microbiota dysbiosis is consistent with other microbiota dysbiosis described to affect the intestinal homeostasis then favouring the development of both inflammation and cancer [30, 47, 48].

In addition to the deleterious role of gut microbiota dysbiosis, our data evidence that perinatal foodborne TiO_2_ exposure is also able to modify the abilities of the intestinal stem cells to survive and grow to generate organoid structures. Moreover, perinatal exposure to foodborne TiO_2_ alters the mRNA level of some markers of CBC and + 4 stem cells as well as the canonical and non-canonical expression of Wnt pathway. These altered mRNA expressions are probably induced and/or linked to the inflammatory context (increased levels of Tnfα, Ifnγ, IL-12 and IL-1β) of the intestinal epithelium perinatally exposed to Tio_2_. Thus, inflammatory cytokines are known to modulate the expression of stem cell markers CBC and + 4, canonical and non-canonical expression of the Wnt pathway and to reduce the organoid growth. For example, the reduced CD44 expression induced by perinatal TiO2 exposure could explain the limited ability of organoids to grow. Indeed, as reduced CD44 expression has been reported to result in loss of CBC [49] and a limited cell proliferation [50], the decreased CD44 expression could explain the limited growth of organoids. At opposite, the increased expression of CD44 (PND 50) could explain the excessive growth of organoids. The inflammatory context of the epithelium could also be involved in the elevated expression of Smoc-2 [51] and, whose increased expression is necessary for increased cell proliferation and motility [52]. Indeed, the expression of Smoc2 is described to be strongly increase during colitis [51]. Finally, the increased expressions of Bim1 and Tert markers by + 4 stem cells evidence the recruitment of these stems cell to support the organoids growth. Again, the inflammatory context is probably the cause of this overexpression, since in inflammatory context, + 4 Stem cells are strongly mobilized to renew the epithelium [53]. Our data also show that perinatal foodborne TiO_2_ exposure increases DNA methylation, as evidenced by elevated 5-Me-dC. This increase of DNA epigenetic marks could participate in the altered abilities of the stem cells to survive, grow and generate a functional epithelium. As described, TiO_2_ can directly modify the DNA methylation in vitro in multiple cell types including into Caco-2 cell line [54], ex vivo in human peripheral blood mononuclear cells [55], as well as in vivo in mouse brain during prenatal exposure [56]. In agreement with these studies, our data evidence that perinatal exposure to foodborne TiO_2_ increase the DNA methylation, with modified levels of 5-Me-dC in colonic epithelial cells. Nevertheless, a recent study has reported that microbiota was able to modulate the epigenetic marks on DNA [57]. In this study, authors have shown that exposure to commensal microbiota induced localized DNA methylation changes at regulatory elements to maintain intestinal homeostasis. Furthermore, microbiota dysbiosis induced by DSS-triggered colitis resulted in profound DNA methylation and chromatin accessibility changes at regulatory elements. Thus, further experiments are required to determine if the modulation of DNA methylation induced by perinatal exposure to TiO_2_ are induced by a direct impact of TiO_2_ or indirectly by the altered microbiota.

In agreement with the numerous in vitro studies reporting that TiO_2_ is able to enhance the secretion of cytokines or chemokines by immune cells including macrophagic, dendritic and lymphocyte cells, our data evidence that perinatal foodborne TiO_2_ exposure alters the expression of both mRNA expression and protein level of cytokines in basal and inflammatory conditions [14]. Moreover, our data evidence that this upregulated cytokine expression is mediated by the gut microbiota dysbiosis induced by perinatal foodborne TiO_2_. Concerning the immune cell composition of the intestinal mucosa, our data are in agreement with the altered immune cells composition of Peyer’s patches following chronic adult exposure to TiO_2_ [21]. In more details, 100 days of TiO_2_ exposure slightly increases the dendritic cell frequency while it reduces the regulatory T-cells in Peyer's patches [21]. In agreement with this study, perinatal foodborne exposure to TiO_2_ strongly increased the frequency of myeloid cells including dendritic and macrophagic cells while it did not affect the T-cells. Finally, perinatal exposure to TiO_2_ strongly reduced the B-cells population into the colonic lamina propria. The consequence of this reduced number of B-cells was the strong decreased level of faecal IgA observed in mice perinatally exposed to foodborne TiO_2_. This reduced B-cells number and IgA response could be driven to the depletion of *Candidatus arthromitus* induced by the exposure to TiO_2_; this bacteria is known to be necessary for inducing the postnatal maturation of gut immune inducing IgA response [46].

Taking together, our findings indicate that both pregnant and lactating women consuming dietary containing TiO_2_ could expose their embryos and baby to an abnormal development and homeostasis of the digestive tract. We demonstrated that the perinatal exposure to foodborne TiO_2_ altered all the compartments of the IBF i.e. microbiota, epithelium and immune system then favouring the development of gut inflammation. Moreover, our data indicated that perinatal exposure to foodborne TiO_2_ also drastically affected the colonic stem cells by reducing their abilities to survive, grow and renew a functional epithelium. Although these reduced abilities of intestinal stem cells are associated with an increased methylation of the cytosine from the DNA, further investigations are needed to determine if perinatal exposure to TiO_2_ can change the methylation of other DNA bases and how perinatal exposure to TiO_2_ changes the DNA imprinting. Thus, additional studies to investigate if the altered imprinting of the DNA is due to a direct impact of TiO_2_ in intestinal stem cells or if it is mediated by the microbiota dysbiosis are needed.

## Conclusions

Our findings demonstrate that environmental factors, exemplified here by TiO_2_ consumption, and subsequent disruption of intestinal homeostasis, might negatively affect the gastrointestinal health and favour the development of chronic intestinal inflammation. Given the high consumption of TiO_2_ worldwide, its ability to translocate across host barrier during the perinatal period and its negative interaction with the gut microbiota to alter the digestive health, these findings are of clear clinical relevance for IBD aetiology and pathophysiology. The interaction between TiO_2_ and the microbiota shown here represents an example of microbiota-food additive interactions. More investigations on how the foodborne TiO_2_ modifies the imprinting of the intestinal stem cells, then altering their abilities to renew a functional epithelium will be of great relevance to better understand the development of the numerous diseases involving a defect in IBF.

## Methods

### Mice

C57BL/6 wild type mice were generated in the animal facility of US006/CREFE. Mice were maintained under 12-h light–dark cycles with free access to food and water. Mice were housed in pathogen-free conditions and pathogen-free conditions were monitored every 6 months in accordance with the full set of FELASA high standard recommendations. Germ-free (GF) C57BL/6 mice were bred and maintained in flexible-film isolators at the Gnotobiology Platform of the Institut Pasteur and at the Clean Mouse Facility of the University of Bern. Sex balance and age matching was ensured in all experimental groups and germ-free status was routinely monitored by culture-dependent and -independent methods. All mouse experiments were performed in accordance with Swiss Federal regulations approved by the Commission for Animal Experimentation of Kanton Bern.

### Study approval

The animal care and ethics committee of US006/CREFE (APAFIS#7496-2016110414017606v6) and 34067_BE35/2022 approved all experimental procedures and they were performed following the guide for the care and use of laboratory animals of the European Council and Canton of Bern.

### Particle dispersion and characterization

TiO_2_ (also known as E171) was obtained from a French supplier of food coloring for bakeries. These particles were fully characterized, as reported in our previous reference [18]. Briefly, mean primary diameter of E171, was 118 ± 53 nm. Their specific surface area was 9.4 m^2^/g and they were > 95% anatase [18]. To deliver to animals, particles were weighted in 15 ml polypropylene vials, and suspended in ultrapure sterile water at a concentration of 54 mg/L. This corresponds to a TiO_2_ intake of 9 mg TiO_2_/kg b.w./day, considering the mice weight to be 30 g and the ingested volume of water to be 5 mL per day. This is in the lower range of the estimated daily exposure of adult humans, which was reported to be between 5.5 and 10.4 mg/kg b.w./day [41]. In this condition, hydrodynamic diameter (Z-average) and polydispersity index (PdI) of E171 were 1078 ± 109 nm (size distribution and transmission electron microscopy images of the particles are provided in Additional file 9: Fig. S9), and their zeta potential was − 2.8 ± 0.2 mV.

### Experimental protocols

Pregnant C57BL/6 wild type female mice were exposed to food additive titanium particles (E171; 9 mg/kg of body weight/day) via drinking water until 4 weeks post-delivery and their offspring was analysed at post-natal day (PND) 30 weaning or maintained under such exposure until PND50. Analyse of both time point allows to address consequences of TiO2 indirect perinatal exposure at PND30 and TiO2 indirect perinatal exposure plus direct exposure at PND50 on IBF.

### Inductively coupled plasma mass spectrometry (ICP-MS)

Acid digestion was performed on dried samples in a DigiPrep heating system, using HNO_3_, NH_4_F, and HClO_4_, as well as H_2_O_2_, with a temperature of 100 °C and several evaporation steps [58]. Digested samples were then diluted in 5% HNO_3_ before analysis on an ICP-MS (iCAP TQ, Thermo Fisher Scientific, United States) along with control samples (blanks with only chemicals and standard reference materials: NRC DOLT-3: dogfish liver, NRC DORM-4: fish protein). Preliminary optimization tests were run to ensure a complete dissolution of TiO_2_ particles during the acidic digestion and to choose the best mode on the ICP-MS machine for Ti analysis (^47^Ti isotope in oxygen mode).

### Model of colitis

To test susceptibility to DSS-induced colitis, adult mice with similar body weights were exposed to two cycles of 2.5% DSS (approximately 40,000 g mol^−1^) in their drinking water (except GF mice that received 1% of DSS), for 7 days, which were interrupted by 7 days of normal water [59]. Control mice received drinking water without DSS. Weight and survival were monitored daily to determine the disease progression. Colitis severity was scored by daily observation of the following parameters: weight loss (0 point, no weight loss or weight gain; 1 point, 5–10% weight loss; 2 points, 11–15% weight loss; 3 points, 16–20% weight loss); stool consistency (0 point, normal and well formed; 2 points, very soft and unformed; 4 points, watery stool); and blood in stool (0 point, normal colour stool; 2 points, reddish colour stool; 4 points, bloody stool). The disease activity index (DAI) was calculated as the combined scores of weight loss, bleeding and stool consistency, with a maximum score of 12. After 21 days, mice were euthanized, the length of the colons was measured and organs, feces and blood were collected for biochemical analysis, and a small piece (0.2 cm) of distal colon was taken for the analysis of gene expression.

### Measurements of paracellular permeability

In vivo permeability assays were performed using fluorescein isothiocyanate (FITC)–dextran 4 kDa (FD4, Sigma) as a paracellular permeability tracer. Mice were gavaged with FD4 (10 mg/100 µL per mice; Sigma) 3 h before the sacrifice [60]. Whole serum FD4 levels were determined with a fluorometer (PerkinElmer, Courtaboeuf, France). Ex vivo permeability assays were done in Ussing chamber systems. After mouse sacrifice, biopsies from colons were mounted in Ussing chamber exposing 0.196cm^2^ of tissue surface to 2.0 ml of circulating oxygenated Ringer solution at 37 °C throughout the experiment. Permeability was assessed by measuring the mucosal-to-serosal flux of FD4 [31].

### Organoid cultures

After the mouse euthanasia, colons were harvested, opened longitudinally and washed in PBS without calcium and magnesium, then transferred to a dissociation medium (PBS, EDTA 9 mM, DTT 3 mM, Y27632 10 µM (final volume 15 ml)). The crypt dissociation was conducted with an orbital spinning wheel for 40 min [61] After discarding the colon residues, the tube contents were centrifuged at 16 G for 5 min at 4 °C [61]. The pellet containing the crypts was washed with 3 mL of DMEM/F12 medium, and sixteen crypts were plated in a 10 µL Matrigel matrix per well (Matrigel, noggin 100 ng/ml, EGF 50 ng/ml, Jagged1 1 µM, CHIR 3 µM). The growth medium was added after a polymerization time of 20 min at 37 °C and was changed every three days [B27 1X, penicillin and streptomycin 100 U/ml, Glutamax 2 mM, N2, R-spondin 0.5 µg/ml, Hepes 1 mM, NAC 0.5 mM, Y27632, noggin 100 ng/ml, EGF 50 ng/ml, Wnt3a medium ½, DMEM/F12 (final volume 1 mL)] [61]. The crypts of each colon were plated in fifteen wells µ-Slide Angiogenesis Ibidi® plate. Organoid stem cell survival (number of organoids formed), and growth capacity (organoid area (µm^2^)) were followed three, six and nine days after plating with a wide field transmission microscope (Apotome Zeiss, 10X lens). The organoid size was monitored by measuring its area using the ImageJ® software, while survival of crypts was studied by counting the number of organoids formed in each well.

### Real-time PCR analysis

Total RNA was extracted from colon tissues using the NucleoSpin RNA II Kit (Macherey–Nagel, France), converted to cDNA using random hexonucleotides, and then used for real-time polymerase chain reaction (RT-PCR) as previously described. Quantitative (q)PCR was performed with QuantiTect SYBR Green PCR Kit [Applied, France] using specific sense and antisense primers (Additional file 11: Table S1). After amplification, threshold cycles (Ct) were determined to obtain expression values in form of 2-∆∆Ct. The investigated genes included: Mucin 2 (*Muc2*), Mucin 3 (*Muc3*), Mucin 4 (*Muc4*), Trefoiled Factor 3 (*TFF3*), Tight junction protein 1 (*Tjp1*), Tight junction protein 2 (*Tjp2*), Occludin (*Ocl*), Claudin 2 (*Cldn2*), Myosin Light Chain Kinase (*Mylk*), CD44, Olfactomedin-4 (*Olfm4*), Leucine-rich repeat-containing G-protein coupled receptor 5 (*Lgr5*), SPARC-related modular calcium-binding protein 2 (*Smoc2*), Achaete scute-like 2 (*Ascl2*), Musashi, telomerase reverse transcriptase (*Tert*), B lymphoma Mo-MLV insertion region 1 homolog (*Bm1*), Homeodomain-only protein X (*Hopx*), *Wnt3A*, *Wnt5A*, Interleukin1β (*Il1b*), Interleukin 6 (*Il6*), Interleukin 10 (*Il10*), Interleukin 12 (*Il12b*), Interleukin 22 (*Il22*), Interleukin (Il23), Tumour necrosis factor α (*Tnfa*), Interferon γ (*Ifng*) and Glyceraldehyde 3-phosphate dehydrogenase (*Gapdh*).

### Flow cytometry

The colon was longitudinally opened, washed and cut into small pieces. Pieces were incubated at RT, three time with buffer consisting of RPMI-1640 medium, 10% fetal calf serum, and 5 mM EDTA on ice. After, pieces were washed with RPMI-1640 medium, 10% calf serum and HEPES 15 mM (Life Technologies) and incubated for 10 min at RT. Colonic tissues were then digested with 0.02% collagenase VIII (Sigma, St Louis, MO, USA) for 50 min at 37 °C under agitation. Supernatant was then passed through a 70-µm cell strainer and centrifuged. A second step of digestion with 0.02% collagenase VIII was performed according to the same modalities. Mononuclear cells were then isolated with 30% Percoll. For cell surface molecular staining, cells were incubated with fluorescent monoclonal antibodies: viability marker FVS440UV-Dapi—BD, CD45 PECF594—BD, TCRβ APC Vio770—Miltenyi, CD11b BuV395—BD, B220 BuV615—BD. Samples were analyzed on a flow cytometer Symphony (BD) and data analyzed with the software FlowJo (Tree Star, Ashland, OR, USA).

### Microbiota analysis by 16S rRNA gene sequencing

Genomic DNA (gDNA) was extracted from colonic mucosa samples by using the Nucleospin Tissue® Kit (Macherey–Nagel®, Düren, Germany) according to the manufacturer’s recommendations. The quantity and quality of the DNA were measured for 16S rDNA sequencing using a Nanodrop spectrophotometer (Thermo Fisher Scientific, Massachusetts, USA), and Qubit®2.0 fluorometer (Thermo Fisher Scientific, Rockford, IL, USA). The 16S rRNA gene was amplified by PCR using universal primers F27 (5′-AGAGTTTGATCMTGGCTCAG-3′) and R1492 (5′-CGGTTACCTTGTTACGACTT-3′). The DNA library was constructed by using the « NEBNext® Ultra™ II FS DNA Library Prep for Illumina» (New England BioLabs). Paired-end sequencing (2 × 150 bp) was performed at Helixio (https://www.helixio.fr, France) on a NextSeq500 (Illumina).

### High-throughput sequencing data analysis

Reads were demultiplexed with bcl2fastq 2.0 software (Illumina), and read quality was assessed using FastQC v0.11.3 (http://www.bioinformatics.babraham.ac.uk/projects/fastqc/). A summary report was constructed with MultiQC [62]. Reads with an average quality score < 20 were trimmed from the ends (5′ and 3′) and the read was removed if its length was less than 100 bp. Following quality control steps, detection of taxa by the k-mer approach was done using Kraken 2 [63] with the SILVA 132 database (https://www.arb-silva.de). The descriptive analyses were performed using phyloseq package in R. Alpha diversity was determined by calculating OTUs number, Chao1, Abundance-based Coverage Estimator (ACE) and Shannon’s diversity index. Differences in gut microbiota beta diversity between groups were visualized using non-metric multidimensional scaling (NMDS) plots with the Bray–Curtis dissimilarity index (Additional file 10: Fig. S10). Differential abundance analysis was then carried out using the edgeR algorithm [64].

### DNA modifications

The levels of 8-oxo-7,8-dihydro-2′-deoxyguanosine (8-oxo-dGuo) and 5-methyl-2′-deoxycytosine (5-Me-dC) were measured by high-performance liquid chromatography–mass spectrometry (HPLC–MS/MS). DNA from frozen colonic crypt pellets (− 80 °C) was extracted using the DNeasy® Blood and Tissue kit (*Qiagen*) according to the manufacturer’s procedure. A RNase treatment step was added after cell lysis, samples were incubated for 2 min with 400 μg of RNaseA. DNA was eluted in 100 μL of 0.1 mM deferoxamine to prevent spurious oxidation. DNA from 50 μL of the sample was digested in two incubation steps. First, the pH was adjusted to 5.5 by adding 5 μL of buffer (100 mM succinic acid, 50 mM calcium chloride, 150 mM, 5 μM zinc sulfate, pH 5.5). Samples were then incubated for 2 h at 37 °C in the presence of 2.5 U of nuclease P1, DNase II, and 0.05 U of phosphodiesterase II. Then, Tris buffer (6 μL, 500 mM Tris, 1 mM ethylenediaminetetraacetic acid, pH 8) was added with 2 U of alkaline phosphatase and 0.05 U of phosphodiesterase I. The sample was incubated for a further 2 h at 37 °C. These samples were neutralized by adding 3.5 µl HCl 0.1 N, filtered on 0.22 µm filter units to eliminate any remaining SiO_2_ particles, and injected onto the high-performance liquid chromatography-tandem mass spectrometry system (HPLC/MS–MS). 8-OxodGuo was quantified with an ExionLC HPLC system connected to a QTRAP 6500 + mass spectrometer (*SCIEX*). The spectrometer was used in the MRM3 mode with positive electrospray ionization. The monitored fragmentation was m/z 284 [M + H]^+^  → 168 [M + H-2-deoxyribose]^+^ → 140 [M + H-2-deoxyribose − CO] + . Chromatographic separations were achieved using a C18 reversed-phase Uptisphere ODB column (*Interchim, Montluçon, France*). The elution was performed using a linear gradient of acetonitrile in 2 mM ammonium formate, starting from 100% ammonium formate and reaching 15% acetonitrile in 30 min at a flow rate of 0.2 mL/min. The retention time was 20 min. In addition to the MS spectrometer, the HPLC eluent was analyzed in a UV detector set at 270 nm to quantify the number of unmodified nucleosides. Levels of 8-oxodGuo were expressed as the number of lesions per million normal bases. The methylation of cytosine was determined in the MRM mode using transitions m/z = 228 [M + H]^+^ → m/z = 112 [M + H-2-deoxyribose]^+^, m/z = 242 [M + H]^+^ → m/z = 142 [M + H-2-deoxyribose]^+^ to quantify 2′-deoxycytidine (dCyd) and 5-MedCyd, respectively, as reported previously [65]. The results are expressed as the percentage of 5-Medcyd relatively to dCyd.

### Humoral response in feces and plasma

Plates were coated with 5 µg/ml of sheep anti-mouse IgA (Sigma) or goat anti-mouse IgG (SouthernBiotech, Cliniscience, Nanterre, France), incubated with plasma, detected with 1.5 µg/ml HRP-conjugated goat anti-mouse IgA (Sigma) or goat anti-mouse IgG (SouthernBiotech). HRP was revealed using TMB and the reaction was stopped with H_2_SO_4_ before reading at 450 nm using Varioskan. microplate reader.

### Lysozyme activity in fecal content

Activity of lysozyme against the peptidoglycan was determined in feces suspended in phosphate-buffered saline using the EnzChek Lysozyme Assay Kit (Molecular probes, Life Technology,St Aubin, France).

### Statistical analysis

Results are expressed as mean ± SEM, except for microbiota. Statistical analyses were performed using GraphPad Prism 9.00 (GraphPad software, San Diego, CA) software package for PC. Multigroup comparisons were performed using a 1-way analysis of variance followed by a Bonferroni correction for multiple tests. Two-group comparisons were performed using an unpaired t test assuming the Gaussian distribution. The Gaussian distribution was tested by Kolmogorov–Smirnov test. A value of P < 0.05 was considered statistically significant. All P values were 2-sided. Significant variations in bacterial population richness and diversity were assessed using Mann and Whitney test. Statistical significances in bacterial population abundance were determined employing two-way analysis of variance with p values < 0.05 considered significant.

### Supplementary Information

**Additional file 1. Fig. S1**: Impact of perinatal exposure to foodborne TiO_2_ on the composition of chemical element of fœtus, spleen and liver from females and pups. (A-C) Wild type female mice have been exposed to TiO_2_ (9 mg/Kg of BW/Day) during the perinatal period including gestational and lactating periods. Then pregnant and lactating females exposed to TiO_2_ or not, have been sacrificed and elemental concentrations have been monitored by ICP-MS in spleen and liver from females (A; end of the weaning days 30), in embryos (B; gestational days 20), and in liver from pup (C; postnatal day 12). Data are expressed as mean ± SEM and were analysed by Student’s t-test. *p < 0.05 vs. control group. At least n = 5 per group.

**Additional file 2. Fig. S2**: Impact of perinatal exposure to foodborne TiO_2_ on the number of pups and male/female ratio. (A and B) Wild type female mice have been exposed to TiO_2_ (9 mg/Kg of BW/Day) during the perinatal period including gestational and lactating periods (A). The number of pups as well as the male ratio per litter have been monitored. Data are expressed as mean ± SEM and were analysed by Student’s t-test. At least n = 8 litters per group.

**Additional file 3. Fig. S3**: Impact of perinatal exposure to foodborne TiO_2_ on colonic microbiota at day 50. (A-E) Wild type female mice have been exposed to TiO_2_ (9 mg/Kg of BW/Day) during the perinatal period including gestational and lactating periods. Weaning pups were also exposed to TiO_2_ (9 mg/Kg of BW/Day) until day 50 after birth (A). Then at day 50 after birth, pups have been sacrificed and the structure of the colonic mucosa-associated microbiota has been monitored by 16S rRNA gene sequencing (B-D). (B) Alpha diversity of colonic mucosal microbiota from exposed or non-exposed mice to foodborne TiO_2_ at day 50 after birth. (C-D) Composition of colonic microbiota at phyla level (C) and Fold changes 2 for bacterial genera significantly perturbed (D) from exposed or non-exposed mice to foodborne TiO_2_ at day and 50 after birth. Data are expressed as median ± SEM and were analysed by Mann and Whitney test. At least n = 8 per group.

**Additional file 4. Fig. S4**: Impact of perinatal exposure to foodborne TiO_2_ on colonic epithelium at day 50. (A-D) Wild type female mice have been exposed to TiO_2_ (9 mg/Kg of BW/Day) during the perinatal period including gestational and lactating periods. Weaning pups were also exposed to TiO_2_ (9 mg/Kg of BW/Day) until day 50 after birth (A-D). Then at day 50 after birth, pups have been sacrificed and several parameters including permeability (A and B), mRNA expression (C and D), abilities of intestinal stem cells to survive (E) and proliferate (F), oxidative stress (G) and DNA methylation (H) were assessed. (A) In vivo permeability was determined by measuring the level of plasmatic FITC-dextran 4 kDa, 3 h following oral administration. (B) Colonic permeability was monitored by measuring the flux of FITC-dextran 4 kDa across colonic biopsies mounted in Ussing chamber for 1 h. (C) mRNA expression of Occludin (*Ocl*), Claudin 2 (*Cldn 2*) Tight junction protein 1 (*Tpj1*) and myosin light chain kinase (*Mlck*) was studied at day 50 after birth. (D) Colonic mRNA expression of CD44, Leucine-rich repeat-containing G-protein coupled receptor 5 (*Lgr5*), Olfactome-din 4 (*Olfm4*), SPARC-related modular calcium-binding protein 2 (*Smoc2*), Achaete-scute complex homolog 2 (*Ascl2*), Musashi RNA-binding protein 1 (*Musashi*), Telomerase reverse transcriptase (*Tert*) and B lymphoma Mo-MLV insertion region 1 homolog (*Bmi1*), homeodomain-only protein homeobox (*Hopx*), canonical (*Wnt3a*) and non-canonical (*Wnt5a*). (E) The organoid survival has been monitored by measuring the percentage of viable organoids according to the time culture. (F) The organoid growth has been studied by measuring the organoid surface according to the time culture. (G) Oxidative stress has been monitored into the epithelial cells from the colonic based crypt of mice perinatally exposed or not to TiO_2_. (H) The quantity of cytosine methylated of DNA from the epithelial cells from the colonic based crypt of mice perinatally exposed or not to TiO_2_. Data are expressed as mean ± SEM and were analysed by Student’s t-test. *p < 0.05; **p < 0.01 and ***p < 0.001 vs control group. At least n = 8 per group.

**Additional file 5. Fig. S5**: Impact of perinatal exposure to foodborne TiO_2_ on mucus, antimicrobial peptides and immunoglobulins. (A-D) Wild type female mice have been exposed to TiO_2_ (9 mg/Kg of BW/Day) during the perinatal period including gestational and lactating periods. Then, at days 30 or 50 after birth, pups have been sacrificed and several parameters including colonic mRNA expression of mucin 2 (*Muc2*), mucin 3 (*Muc3*), mucin 4 (*Muc4*) and Trefoiled factor 3 (*Tff3*) (A, B), faecal levels of lysozym (C) and IgG (D). Data are expressed as mean ± SEM and were analysed by Student’s t-test. *p < 0.05 and ***p < 0.001 vs. control group. At least n = 5 per group.

**Additional file 6. Fig. S6**: Impact of perinatal exposure to TiO_2_ foodborne on intestinal immune system. (A-C) Wild type female mice have been exposed to TiO_2_ (9 mg/Kg of BW/Day) during the perinatal period including gestational and lactating periods. Weaning pups were also exposed to TiO_2_ (9 mg/Kg of BW/Day) until day 50 after birth. Then, at days 30 or 50 after birth, pups have been sacrificed and several parameters including mRNA expression have been monitored. (A–C) On Peyer’s patches (A) and scrapped ileal (B) and colon (C)), mRNA expression of *Il1b*, *Il6*, *Il10, Il12*, *Il22, Il23*, *TNFa*, *IFNg*, was monitored at days 30 and 50 after birth. Data are expressed as mean ± SEM and were analysed by Student’s t-test. *p < 0.05 and **p < 0.01 vs. control group. At least n = 8 per group.

**Additional file 7. Fig. S7**: Impact of perinatal exposure to foodborne TiO2 on cytokines mRNA expression in context of colitis. Wild type female mice have been exposed to TiO_2_ (9 mg/Kg of BW/Day) during the perinatal period including gestational and lactating periods. At 14 weeks of age, colitis has been orally induced by introducing Dextran Sulfate Sodium (DSS) into drinking water at 2% for 7 days followed by 7 days of regular water then 7 days of DSS. Then, at the end of the DSS procedure, mice have been sacrificed and cytokine mRNA expression have been monitored. Data are expressed as mean ± SEM and were analysed by Student’s t-test. **p < 0.01; ***p < 0.001 and ****p < 0.0001 vs. control group. At least n = 5 per group.

**Additional file 8. Fig. S8**: Impact of gut microbiota dysbiosis induced by perinatal exposure to TiO_2_ on the cytokines mRNA expression in context of colitis. Germ free mice female have been exposed to gut microbiota dysbiosis induced by perinatal foodborne until the weaning i.e. postnatal day 30. At 14 weeks of age, colitis has been orally induced by introducing Dextran Sulfate Sodium (DSS) into drinking water at 2% for 7 days followed by 7 days of regular water then 7 days of DSS. Then, at the end of the DSS procedure, mice have been sacrificed and cytokines mRNA expression have been monitored. Data are expressed as mean ± SEM and were analysed by Student’s t-test. *p < 0.05; **p < 0.01; ***p < 0.001 and ****p < 0.0001 vs. control group. At least n = 5 per group.

**Additional file 9. Fig. S9**: Physico-chemical characteristics of TiO_2_ particles. (A) Transmission electron microscopy images of TiO_2_ particles, recorded on a JEOL 1200EX TEM operating at 80 kV (Grenoble Institut des Neurosciences, Grenoble, France). (B) Size distribution of TiO_2_ particles in the drinking water, measured via dynamic light scattering on a Malvern nanoZS zetasizer.

**Additional file 10. Fig. S10**: Beta diversity analysis of colonic microbiota. Beta diversity of the colonic bacterial community (all bacterial taxa considered) was analyzed using multidimensional non-metric scaling (NMDS) plots generated by Bray–Curtis dissimilarity index. The four mouse groups are represented by different colors: pink for mice non-exposed at TiO_2_ at days 30, green for mice non-exposed at TiO_2_ at days 50, blue mice exposed at TiO_2_ at days 30 and purple for exposed at TiO_2_ at days 50.

**Additional file 11. Table S1**: Sequence of oligonucleotides used for RT-qPCR experiments.
