# Correction: Perinatal foodborne titanium dioxide exposure-mediated dysbiosis predisposes mice to develop colitis through life

**DOI:** 10.1186/s12989-024-00570-0

**Published:** 2024-03-06

**Authors:** Caroline Carlé, Delphine Boucher, Luisa Morelli, Camille Larue, Ekaterina Ovtchinnikova, Louise Battut, Kawthar Boumessid, Melvin Airaud, Muriel Quaranta-Nicaise, Jean-Luc Ravanat, Gilles Dietrich, Sandrine Menard, Gérard Eberl, Nicolas Barnich, Emmanuel Mas, Marie Carriere, Ziad Al Nabhani, Frédérick Barreau

**Affiliations:** 1 Institut de Recherche en Santé Digestive (IRSD), INSERM UMR-1220, Purpan Hospital, CS60039, University of Toulouse, INSERM, INRAE, ENVT, UPS, 31024 Toulouse Cedex 03, France; 2 M2iSH, Université Clermont Auvergne, UMR1071 INSERM, USC INRAE 1382, Clermont-Ferrand, France; 3 Department of Visceral Surgery and Medicine, Bern University Hospital, University of Bern, 3010 Bern, Switzerland; 4 Maurice Müller Laboratories, Department for Biomedical Research, University of Bern, 3008 Bern, Switzerland; 5 Laboratoire Ecologie Fonctionnelle et Environnement, Université de Toulouse, CNRS, Toulouse, France; 6 Univ. Grenoble-Alpes, CEA, CNRS, IRIG-SyMMES, CIBEST, Grenoble, France; 7 Institut Pasteur, Microenvironment and Immunity Unit, 75724 Paris, France; 8 INSERM U1224, Paris, France; 9 Gastroenterology, Hepatology, Nutrition, Diabetology and Hereditary Metabolic Diseases Unit, Hôpital des Enfants, CHU de Toulouse, 31300 Toulouse, France

**Correction: Particle and Fibre Toxicology (2023) 20:45** 10.1186/s12989-023-00555-5

Following publication of the original article [1], the authors reported some spelling and bibliograph errors. Below is a table of corrections which have been implemented in the original article.

The original article [1] has been corrected.

**Table:** 

Section	Originally published text	Corrected text
Abstract	Perinatal exposure to titanium dioxide (TiO_2_), as a foodborne particle, may influence the intestinal barrier function and the susceptibility to develop inflammatory bowel disease (IBD) later in life	Perinatal exposure to titanium dioxide (TiO_2_), as a foodborne particle, may influence the intestinal barrier function and the susceptibility to develop inflammatory bowel diseases (IBD) later in life
Background	A significant number of human chronic diseases (inflammatory, metabolic …) is linked to a deficiency of the IBF and some of them, like IBD, exhibit alterations of the four IBF’s compartments [8, 9]	significant number of human chronic diseases (inflammatory, metabolic …) is linked to a deficiency of the IBF and some of them, like IBD, exhibit alterations of the three IBF’s compartments [8, 9]
	To evaluate this hypothesis, we exposed pregnant female C57BL/6 mice to 9 mg E171/kg b.w./day via their drinking water,from the beginning of gestation until 3 weeks postdelivery	To evaluate this hypothesis, we exposed pregnant female C57BL/6 mice to 9 mg E171/kg b.w./day via their drinking water, from the beginning of gestation until 4 weeks postdelivery
	This exposure concentration is in the lower range of the estimated daily exposure of human adults, which ranges between 5.5 and 10.4 mg/kg b.w./day according to EFSA’s estimations [ref 35]	This exposure concentration is in the lower range of the estimated daily exposure of human adults, which ranges between 5.5 and 10.4 mg/kg b.w./day according to EFSA’s estimations [29]
	When considering the guidances on dose conversion between human and animal exposure, such as the Nair and Jacob practice guide or FDA’s guidelines, we previously estimated that doses up to 50–60 mg/kg b.w./day in mice would be realistic [ref notre revue PFT] confirming that the dose used in the present study can be considered as a low exposure dose	When considering the guidances on dose conversion between human and animal exposure, such as the Nair and Jacob practice guide or FDA’s guidelines, we previously estimated that doses up to 50–60 mg/kg b.w./day in mice would be realistic [14] confirming that the dose used in the present study can be considered as a low exposure dose
Results	**Figure 1** Abilities of foodborne TiO_2_ to translocate across the human barriers. A–G Wild type female mice have been exposed to TiO_2_ (9 mg/BW/Day)	**Figure 1** Abilities of foodborne TiO_2_ to translocate across the human barriers. A–G Wild type female mice have been exposed to TiO_2_ (9 mg/Kg ofBW/Day)
	Since gut microbiota is described to modulate the intestinal epithelium homeostasis [29, 30], we investigated if perinatal exposure to foodborne TiO_2_	Since gut microbiota is described to modulate the intestinal epithelium homeostasis [30, 31], we investigated if perinatal exposure to foodborne TiO_2_
	In addition, the expression of myosin light chain kinase (*Mylk*), a master regulator of the tight junction opening [31], was increased by perinatal exposure	In addition, the expression of myosin light chain kinase (*Mylk*), a master regulator of the tight junction opening [32], was increased by perinatal exposure
	**Figure 2** Impact of perinatal exposure to foodborne TiO_2_ on colonic microbiota at days 30. A**-E** Wild type female mice have been exposed to TiO_2_ (9 mg/BW/Day) during the perinatal period including gestational and lactating periods. Then at days 30 after birth, pups have been sacrificed and the structure of the colonic mucosa‑associated microbiota has been monitored by 16S rRNA gene sequencing (B**-E**) C-E Composition of colonic microbiota at phyla level (C) and Fold changes 2 for bacterial genera significantly perturbed (D and E) from exposed or non‑exposed mice to foodborne TiO_2_ at day 30 after birth	**Figure 2** Impact of perinatal exposure to foodborne TiO_2_ on colonic microbiota at day 30. A**-D** Wild type female mice have been exposed to TiO_2_ (9 mg/Kg of BW/Day) during the perinatal period including gestational and lactating periods. Then at day 30 after birth, pups have been sacrificed and the structure of the colonic mucosa‑associated microbiota has been monitored by 16S rRNA gene sequencing (B**-D**) C-D Composition of colonic microbiota at phyla level (C) and Fold changes 2 for bacterial genera significantly perturbed (D) from exposed or non‑exposed mice to foodborne TiV at day 30 after birth
	At days 50 after birth, TiO_2_ exposure only increased the level of *Muc2* (Additional file 5: Fig.S5A, B)	At days 50 after birth, TiO_2_ exposure only increased the level of *Muc2* (Additional file 5: Fig. S5A-C)
	At days 50 after birth, TiO_2_ exposure only increased the level of Muc2 (Additional file 5: Fig. S5A, E)	At days 50 after birth, TiO_2_ exposure only increased the level of Muc2 (Additional file 5: Fig. S5A)
	Since perinatal exposure to TiO_2_ altered the func-tionality of the colonic epithelium, we then monitored its effects on the intestinal epithelial stem cells (IESC) homeostasis (Fig. 3D–F; Additional file 5: Fig. S3D–F)	Since perinatal exposure to TiO_2_ altered the func-tionality of the colonic epithelium, we then monitored its effects on the intestinal epithelial stem cells (IESC) homeostasis (Fig. 4D–F; Additional file 5: Fig. S4D–F)
	At day 50, mice exposed to TiO_2_ had an increased mRNA levels of colonic CD44, Leucine-rich repeat-containing G-protein coupled receptor 5 (*Lgr5*), Achaete-scute complex homolog 2 (*Ascl2*) and Musashi RNA-binding protein 1 (*Musashi*), three markers of CBC, Telomerase reverse transcriptase (*Tert*) and Homeodomain-only protein X (*Hopx*), two markers of + 4 stem cells and the marker of non-canonical wnt pathway (wnt5, involved in inflammatory pathway) (Additional file 3: Fig. S3D) but	At day 50, mice exposed to TiO_2_ had an increased mRNA levels of colonic CD44, Leucine-rich repeat-containing G-protein coupled receptor 5 (*Lgr5*), Achaete-scute complex homolog 2 (*Ascl2*) and Musashi RNA-binding protein 1 (*Musashi*), three markers of CBC, Telomerase reverse transcriptase (*Tert*) and Homeodomain-only protein X (*Hopx*), two markers of + 4 stem cells and the marker of non-canonical wnt pathway (wnt5, involved in inflammatory pathway) (Additional file 4: Fig. S4D) but
	**Figure 3** Impact of perinatal exposure to foodborne TiO_2_ on colonic epithelium at day 30. A–D Wild type female mice have been exposed to TiO_2_ (9 mg/BW/Day)	**Figure 3** Impact of perinatal exposure to foodborne TiO_2_ on colonic epithelium at day 30. A–D Wild type female mice have been exposed to TiO_2_ (9 mg/Kg of BW/Day)
	We observed a significant reduction of organoid growth at day 9 post-organoid culture obtained from TiO_2_-exposed mice compared to control at day 30 (Fig. 3E) but the survival of colonic organoids was similar between both TiO_2_-treated and untreated group (Fig. 3F)	We observed a significant reduction of organoid growth at day 9 post-organoid culture obtained from TiO_2_-exposed mice compared to control at day 30 (Fig. 3F) but the survival of colonic organoids was similar between both TiO_2_-treated and untreated group (Fig. 3E)
	Finally, since oxidative stress and/or DNA meth-ylation are well known to regulate gene expression, we monitored the impact of exposure to TiO_2_ on the oxida-tive balance as well as DNA methylation of the colonic epithelium (Fig. 3G, H; Additional file 4: Fig. S4H)	Finally, since oxidative stress and/or DNA meth-ylation are well known to regulate gene expression, we monitored the impact of exposure to TiO_2_ on the oxida-tive balance as well as DNA methylation of the colonic epithelium (Fig. 3G, H; Additional file 4: Fig. S4G)
	In this objective, we used 8-oxo-dGuo as a biomarker of DNA oxidation, this lesion being also considered as a marker of oxidative stress [32] and being quantifiable with a high sensitivity using methods such as HPLC-tandem mass spectrometry [33]	In this objective, we used 8-oxo-dGuo as a biomarker of DNA oxidation, this lesion being also considered as a marker of oxidative stress [33] and being quantifiable with a high sensitivity using methods such as HPLC-tandem mass spectrometry [34]
	As a DNA methylation biomarker, we quantified 5-methyl-2′-deoxycitidine, i.e., 5-Me-dC, as it is the predominant methylation site in mammalian genomes and it shows the highest biological significance as it modulates the binding of transcription factors to DNA [34, 35]	As a DNA methylation biomarker, we quantified 5-methyl-2′-deoxycitidine, i.e., 5-Me-dC, as it is the predominant methylation site in mammalian genomes and it shows the highest biological significance as it modulates the binding of transcription factors to DNA [29, 35]
	**Figure 4** Impact of perinatal exposure to TiO_2_ foodborne on intestinal immune system. A**–E** Wild type female mice have been exposed to TiO_2_O_2_ (9 mg/BW/Day) during	**Figure 4** Impact of perinatal exposure to TiO_2_ foodborne on intestinal immune system. A**–D** Wild type female mice have been exposed to TiO_2_ (9 mg/Kg of BW/Day) during
	In contrast to those observed in colon of young mice, perinatal exposure to TiO_2_ did not affect the mRNA level of *Il23* while it increased the expression of *Il1b*, *Il6*, *Il10*, *Il22 and Tnfa* (Additional file 6: Fig. S6C)	In contrast to those observed in colon of young mice, perinatal exposure to TiO_2_ did not affect the mRNA level of *Il23* at day 50 while it increased the expression of *Il1b*, *Il6*, *Il10*, *Il22, Tnfa and Ifng* (Additional file 6: Fig. S6C)
	However, at protein level, perinatal exposure to TiO_2_ increased the colonic cytokines expression of Tnfα, Ifnγ, IL-12 and IL-1β (Fig. 4A)	However, at protein level, perinatal exposure to TiO_2_ increased the colonic cytokines expression of Tnfα, Ifnγ, IL-12 and IL-1β (Fig. 4A) at day 30
	Regarding colonic immune cell populations, flow cytometry experiments on the lamina propria from colon of mice (day 50) evidenced that perinatal exposure to TiO_2_ increased the percentage of myeloid cells (CD11^+^),	Regarding colonic immune cell populations, flow cytometry experiments on the lamina propria from colon of mice (day 50) evidenced that perinatal exposure to TiO_2_ increased the percentage of myeloid cells (CD11b^+^),
	Finally, the reduced percentage of B cells in the lamina propria was associated with reduced faecal levels of IgA, but not IgG at both days 30 and 50 after birth (Fig. 4D; Additional file 5: Fig. S5D)	Finally, the reduced percentage of B cells in the lamina propria was associated with reduced faecal levels of IgA, but not IgG at both days 30 and 50 after birth (Fig. 4B–D; Additional file 5: Fig. S5D)
	Since gut microbiota dysbiosis has been shown to alter the gut homeostasis [7, 29, 38],	Since gut microbiota dysbiosis has been shown to alter the gut homeostasis [7, 30, 38],
	Six weeks after microbiota transfer, permeability and mRNA levels of *Occludin*, *Tpj1*, *Tpj2* and *Mylk* as well as *Il1b*, *Il12*, *Tnfa* and *Ifng* were assessed (Fig. 5B, C). As	Six weeks after microbiota transfer, permeability and mRNA levels of *Occludin*, *Tpj1*, *Tpj2* and *Mylk* as well as *Il1b*, *Il12*, *Tnfa* and *Ifng* were assessed (Fig. 5B–D). As
	As illustrated in Fig. 5B, the transfer of T iO_2_-triggered microbiota dysbiosis to healthy germ-free mice led to significantly increased paracellular intestinal permeability (Fig. 5B), increased mRNA level of *Mylk*, and reduced mRNA level of *Tjp1* and Tjp2 (Fig. 5C)	As illustrated in Fig. 5B, the transfer of T iO_2_-triggered microbiota dysbiosis to healthy germ-free mice led to significantly increased paracellular intestinal permeability (Fig. 5B), increased mRNA level of *Mylk*, and reduced mRNA level of *Tjp1* and Tjp2 (Fig. 5C) in offspring at day 30
	We observed that alteration of homeostasis of the colonic mucosa related to early life exposure to TiO_2_O_2_ did not persist until adult 17 weeks of age as monitored for permeability, cytokine and other inflammatory markers i. e. in the group unchallenged for DSS mice exposed to TiO_2_ superpose with mice unexposed (Fig. 6; Additional file 7: Fig. S7A)	We observed that alteration of homeostasis of the colonic mucosa related to early life exposure to TiO_2_ did not persist until adult 17 weeks of age as monitored for permeability, cytokine and other inflammatory markers i. e. in the group unchallenged for DSS mice exposed to TiO_2_ superpose with mice unexposed (Fig. 6; Additional file 7: Fig. S7)
	However, as illustrated in Fig. 6B–H, perinatal exposure to TiO_2_ enhanced significantly the loss of body weight and the DAI induced by DSS	However, as illustrated in Fig. 6B–G, perinatal exposure to TiO_2_ enhanced significantly the loss of body weight and the DAI induced by DSS. Perinatal
	Figure 6 Impact of perinatal exposure to foodborne TiO_2_ on susceptibility to develop colitis later in life. A–G Wild type female mice have been exposed to TiO_2_ (9 mg/BW/Day) during the perinatal period including gestational and lactating periods (A)	Figure 6 Impact of perinatal exposure to foodborne TiO_2_ on susceptibility to develop colitis later in life. A–G Wild type female mice have been exposed to TiO_2_ (9 mg/Kg of BW/Day) during the perinatal period including gestational and lactating periods (A)
	Perinatal exposure to TiO_2_ also exacerbated the colitis, as evidenced by a reduced colon length associated with increased colonic mRNA expression and protein levels of IL-1β, IL-4, IL-12, IL-13, IFNγ and TNF-α (Additional file 6: Fig. S6A and additional File 7: FigS7E)	Perinatal exposure to TiO_2_ also exacerbated the colitis, as evidenced by a reduced colon length associated with increased colonic mRNA expression and protein levels of IL-1β, IL-4, IL-12, IL-13, IFNγ and TNF-α (Additional file 7: Fig. S7)
	Perinatal exposure to TiO_2_ also aggravated significantly the alterations of intestinal permeability, as evidenced by an increased Dextran-FITC flux, mRNA expression of MLCK and a reduced mRNA level of Tjp1 (Fig. 6G)	Perinatal exposure to TiO_2_ also aggravated significantly the alterations of intestinal permeability, as evidenced by an increased 4 kDa Dextran-FITC flux, mRNA expression of MLCK and a reduced mRNA level of Tjp1 (Fig. 6G)
	In contrast, at the 17th week of life, there was no longer any signifi-cant difference in terms of permeability, cytokine or other inflammatory markers i. e. in the group unchallenged for DSS mice exposed to TiO_2_ superpose with mice unex-posed (Fig. 7D–H)	In contrast, at the 17th week of life, there was no longer any signifi-cant difference in terms of permeability, cytokine or other inflammatory markers i. e. in the group unchallenged for DSS mice exposed to TiO_2_ superpose with mice unex-posed (Fig. 7E–G)
	The colitis was exacerbated in these animals, as evidenced by a reduced colon length associated with increased colonic mRNA expression and protein levels of IL-1β, IL-4, IL-12, IL-13, IFNγ and TNF-α (Additional file 8: Fig. S8 A and Additional file 7: Fig. S7E)	The colitis was exacerbated in these animals, as evidenced by a reduced colon length associated with increased colonic mRNA expression and protein levels of IL-1β, IL-4, IL-12, IL-13, IFNγ and TNF-α (Additional file 8: Fig. S8 and file 7: Fig. 7E)
Discussion	In this study, authors evidenced that foodborne TiO_2_ parti-cles were able to cross the cotyledon of human placenta while no data are available concerning their potential in vivo passage [42] Moreover, the presence of Ti in the meconium do not indicate if its passage underwent dur-ing gestation and/or the beginning of suckling	In this study, authors evidenced that foodborne TiO_2_ parti-cles were able to cross the cotyledon of human placenta while no data are available concerning their potential in vivo passage [42]. Moreover, the presence of Ti in the meconium does not indicate if its passage underwent dur-ing gestation and/or the beginning of suckling
	This bacteria, which resides in the intestinal mucus layer har-bors some virulence traits (type VI secretion system and putative effector proteins) [43], which can trigger CD-like disease in the presence of impaired clearance of the bac-terium by innate immunity [44]	This bacteria, which resides in the intestinal mucus layer har-bors some virulence traits (type VI secretion system and putative effector proteins) [43], which can trigger IBD-like disease in the presence of impaired clearance of the bac-terium by innate immunity [44]
	The deleterious impact of this microbiota dysbiosis is consistent with other microbiota dysbiosis described to affect the intestinal homeostasis then favouring the development of both inflammation and cancer [29, 47, 48]	The deleterious impact of this microbiota dysbiosis is consistent with other microbiota dysbiosis described to affect the intestinal homeostasis then favouring the development of both inflammation and cancer [30, 47, 48]
	hese altered mRNA expressions are probably induced and/or linked to the inflammatory context (increased levels of Tnfα, Ifnγ, IL-12 and IL-1β) of the intestinal epithelium perina-tally exposed to TiO_2_	hese altered mRNA expressions are probably induced and/or linked to the inflammatory context (increased levels of Tnfα, Ifnγ, IL-12 and IL-1β) of the intestinal epithelium perina-tally exposed to TiO_2_
	Nevertheless, a recent study has reported that microbiota was able to modulate the epigenic marks on DNA [57]	Nevertheless, a recent study has reported that microbiota was able to modulate the epigenetic marks on DNA [57]
	In more details, 100 days of TiO_2_ exposure slightly increase the dendritic cell frequency while it reduces the regulatory T-cells in Peyer’s patches [21]	In more details, 100 days of TiO_2_ exposure slightly increases the dendritic cell frequency while it reduces the regulatory T-cells in Peyer’s patches [21]
Methods	Pregnant C57BL/6 wild type female mice were exposed to food additive titanium particles (E171; 9 mg/kg of body weight/day) via drinking water until 3 weeks post-delivery and their offspring was analysed at post-natal day (PND) 30 weaning or maintained under such expo-sure until PND50	Pregnant C57BL/6 wild type female mice were exposed to food additive titanium particles (E171; 9 mg/kg of body weight/day) via drinking water until 4 weeks post-delivery and their offspring was analysed at post-natal day (PND) 30 weaning or maintained under such expo-sure until PND50
	Mice were gavaged with FD4 (10 mg/100 µL per mice; Sigma) 4 h before the sacrifice [ 60]	Mice were gavaged with FD4 (10 mg/100 µL per mice; Sigma) 3 h before the sacrifice [ 60]
	Permeability was assessed by measuring the mucosal-to-serosal flux of FD4 [30]	Permeability was assessed by measuring the mucosal-to-serosal flux of FD4 [31]
	Organoid stem cell survival (number of organoids formed), and growth capacity (organoid area (µm^2^)) were followed three, six, nine and twelve days after plating with a wide field transmission microscope (Apotome Zeiss, 10X lens)	Organoid stem cell survival (number of organoids formed), and growth capacity (organoid area (µm^2^)) were followed three, six and nine days after plating with a wide field transmission microscope (Apotome Zeiss, 10X lens)
Supplementary Information	Additional file 1. Fig. S1: Impact of perinatal exposure to foodborne TiO_2_O_2_ on the composition of chemical element of fœtus, spleen and liver from females and pups. (A‑C) Wild type female mice have been exposed to TiO_2_ (9 mg/BW/Day)	Additional file 1. Fig. S1: Impact of perinatal exposure to foodborne TiO_2_ on the composition of chemical element of fœtus, spleen and liver from females and pups. (A‑C) Wild type female mice have been exposed to TiO_2_ (9 mg/Kg of BW/Day)
	(A and B) Wild type female mice have been exposed to TiO_2_ (9 mg/BW/Day) during the perinatal period including gestational and lactating periods	(A and B) Wild type female mice have been exposed to TiO_2_O_2_ (9 mg/Kg of BW/Day) during the perinatal period including gestational and lactating periods
	A‑E) Wild type female mice have been exposed to TiO_2_ (9 mg/BW/Day) during the perinatal period including gestational and lactating periods	A‑E) Wild type female mice have been exposed to TiO_2_ (9 mg/Kg of BW/Day) during the perinatal period including gestational and lactating periods
	Weaning pups were also exposed to TiO_2_ (9 mg/BW/Day) until day 50 after birth (A)	Weaning pups were also exposed to TiO_2_ (9 mg/Kg of BW/Day) until day 50 after birth (A)
	Then at day 50 after birth, pups have been sacrificed and the structure of the colonic mucosa‑associ‑ ated microbiota has been monitored by 16S rRNA gene sequencing (B‑E)	Then at day 50 after birth, pups have been sacrificed and the structure of the colonic mucosa‑associ‑ ated microbiota has been monitored by 16S rRNA gene sequencing (B‑D)
	(C‑E) Composition of colonic microbiota at phyla level (C) and Fold changes 2 for bacterial genera significantly perturbed (D and E) from exposed or non‑exposed mice to foodborne TiV at day and 50 after birth	C‑D) Composition of colonic microbiota at phyla level (C) and Fold changes 2 for bacterial genera significantly perturbed (D) from exposed or non‑exposed mice to foodborne TiO_2_ at day and 50 after birth
	Additional file 4. Fig. S4: Impact of perinatal exposure to foodborne TiO_2_ on colonic epithelium at day 50. (A‑D) Wild type female mice have been exposed to TiO_2_ (9 mg/BW/Day) during the perinatal period including ges‑ tational and lactating periods. Weaning pups were also exposed to TiO_2_ (9 mg/BW/Day) until day 50 after birth (A‑D)	Additional file 4. Fig. S4: Impact of perinatal exposure to foodborne TiO_2_ on colonic epithelium at day 50. (A‑D) Wild type female mice have been exposed to TiO_2_ (9 mg/Kg of BW/Day) during the perinatal period including ges‑ tational and lactating periods. Weaning pups were also exposed to TiO_2_ (9 mg/Kg of BW/Day) until day 50 after birth (A‑D)
	(A‑D) Wild type female mice have been exposed to T iO_2_ (9 mg/BW/Day) during the perinatal period including gestational and lactating periods. Then, at days 30 or 50 after birth, pups have been sacrificed and several parameters including colonic mRNA expression of mucin 2 (*Muc2*), mucin 3 (*Muc3*),mucin 4 (*Muc4*) and Trefoiled factor 3 (*Tff3*) (A), faecal levels of lysozyme (B) and IgG (C)	(A‑D) Wild type female mice have been exposed to T iO_2_ (9 mg/Kg of BW/Day) during the perinatal period including gestational and lactating periods. Then, at days 30 or 50 after birth, pups have been sacrificed and several parameters including colonic mRNA expression of mucin 2 (*Muc2*), mucin 3 (*Muc3*), mucin 4 (*Muc4*) and Trefoiled factor 3 (*Tff3*) (A, B), faecal levels of lysozyme (C) and IgG (D)
	(A‑C) Wild type female mice have been exposed to TiO_2_ (9 mg/BW/Day) during the perinatal period including gestational and lactating periods. Weaning pups were also exposed to TiO_2_ (9 mg/BW/Day) until day 50 after birth	(A‑C) Wild type female mice have been exposed to TiO_2_ (9 mg/Kg of BW/Day) during the perinatal period including gestational and lactating periods. Weaning pups were also exposed to TiO_2_ (9 mg/Kg of BW/Day) until day 50 after birth
	Wild type female mice have been exposed to TiO_2_ (9 mg/BW/Day) during the perinatal period including gestational and lactating periods. A	Wild type female mice have been exposed to TiO_2_ (9 mg/Kg of BW/Day) during the perinatal period including gestational and lactating periods. A
