# Supplementary figures and images for: Perinatal foodborne titanium dioxide exposure-mediated dysbiosis predisposes mice to develop colitis through life
Source: Part Fibre Toxicol. 2023 Nov 23;20:45. doi: 10.1186/s12989-023-00555-5 (PMC10666382; doi:10.1186/s12989-023-00555-5)

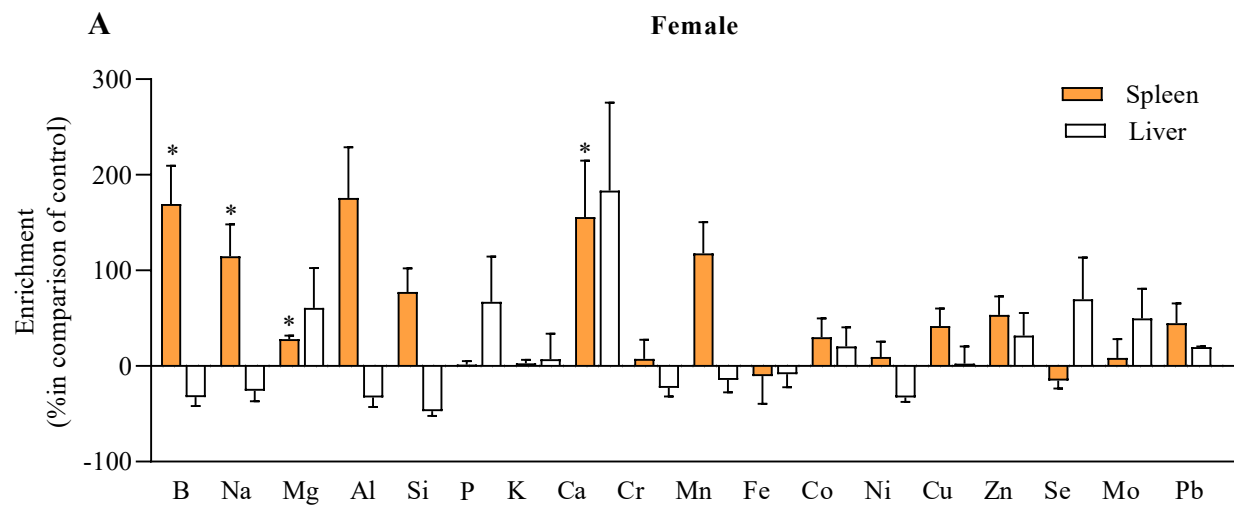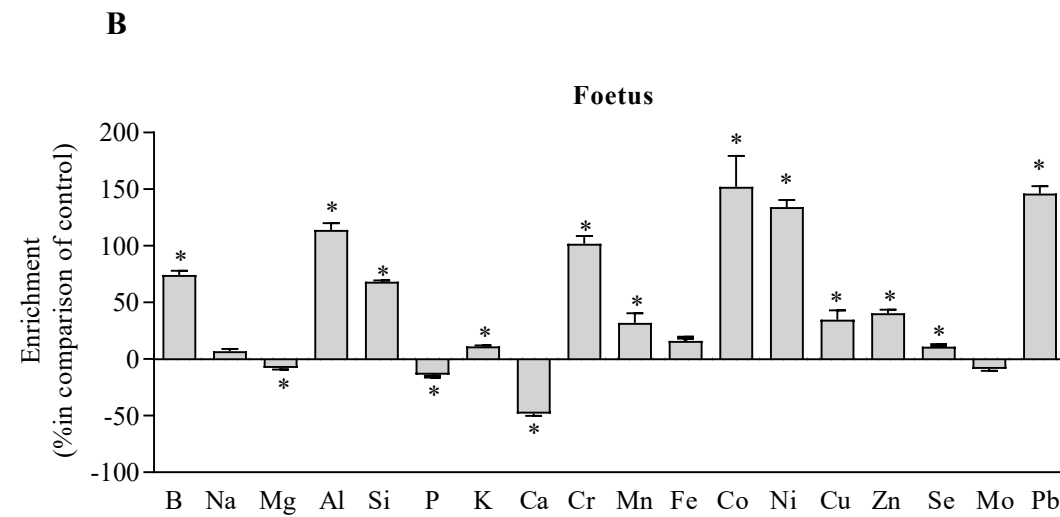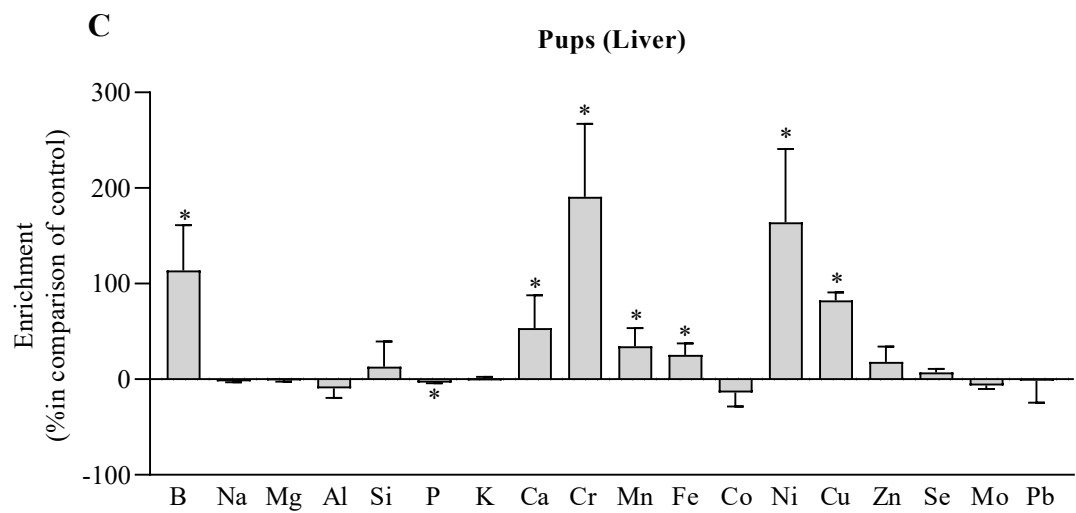

Supplement: Supplementary file 1 — Additional file 1. Fig. S1: Impact of perinatal exposure to foodborne TiO2 on the composition of chemical element of fœtus, spleen and liver from females and pups. (A-C) Wild type female mice have been exposed to TiO2 (9 mg/Kg of BW/Day) during the perinatal period including gestational and lactating periods. Then pregnant and lactating females exposed to TiO2 or not, have been sacrificed and elemental concentrations have been monitored by ICP-MS in spleen and liver from females (A; end of the weaning days 30), in embryos (B; gestational days 20), and in liver from pup (C; postnatal day 12). Data are expressed as mean ± SEM and were analysed by Student’s t-test. *p < 0.05 vs. control group. At least n = 5 per group. [file 12989_2023_555_MOESM1_ESM.pdf]

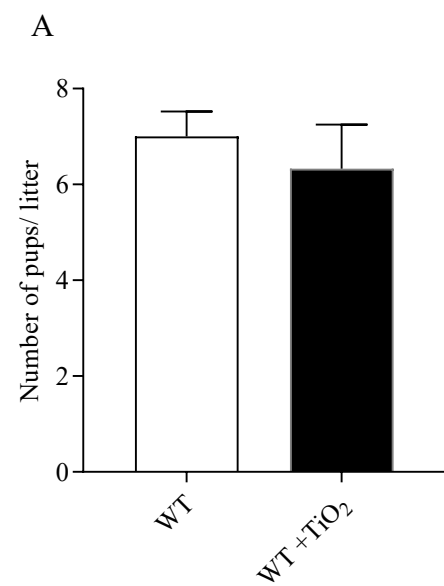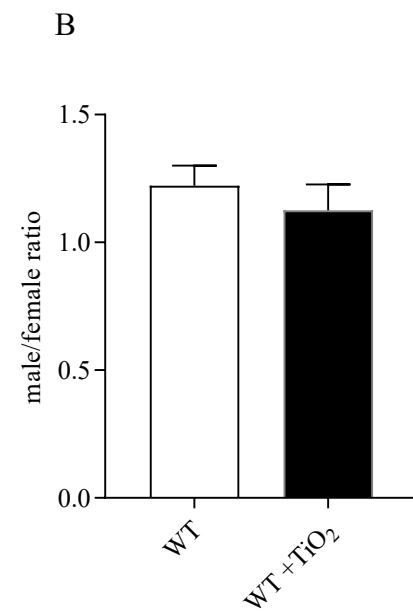

Supplement: Supplementary file 2 — Additional file 2. Fig. S2: Impact of perinatal exposure to foodborne TiO2 on the number of pups and male/female ratio. (A and B) Wild type female mice have been exposed to TiO2 (9 mg/Kg of BW/Day) during the perinatal period including gestational and lactating periods (A). The number of pups as well as the male ratio per litter have been monitored. Data are expressed as mean ± SEM and were analysed by Student’s t-test. At least n = 8 litters per group. [file 12989_2023_555_MOESM2_ESM.pdf]

A

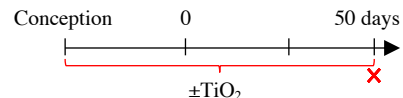

B

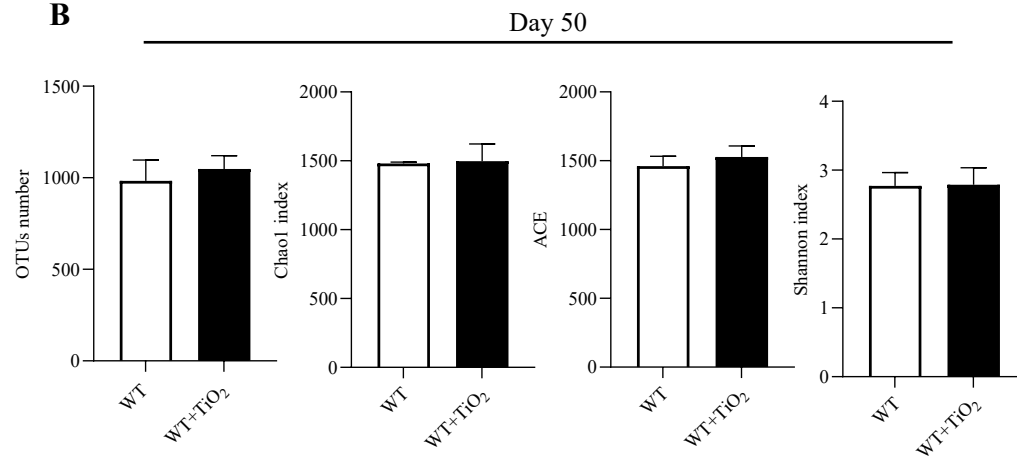

C

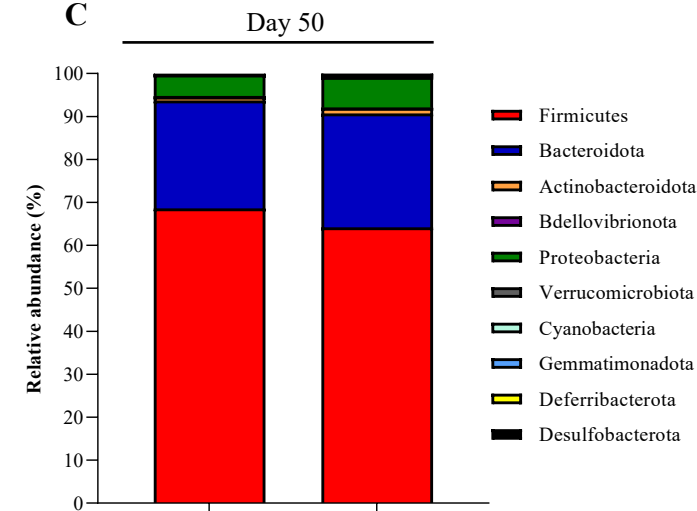

D

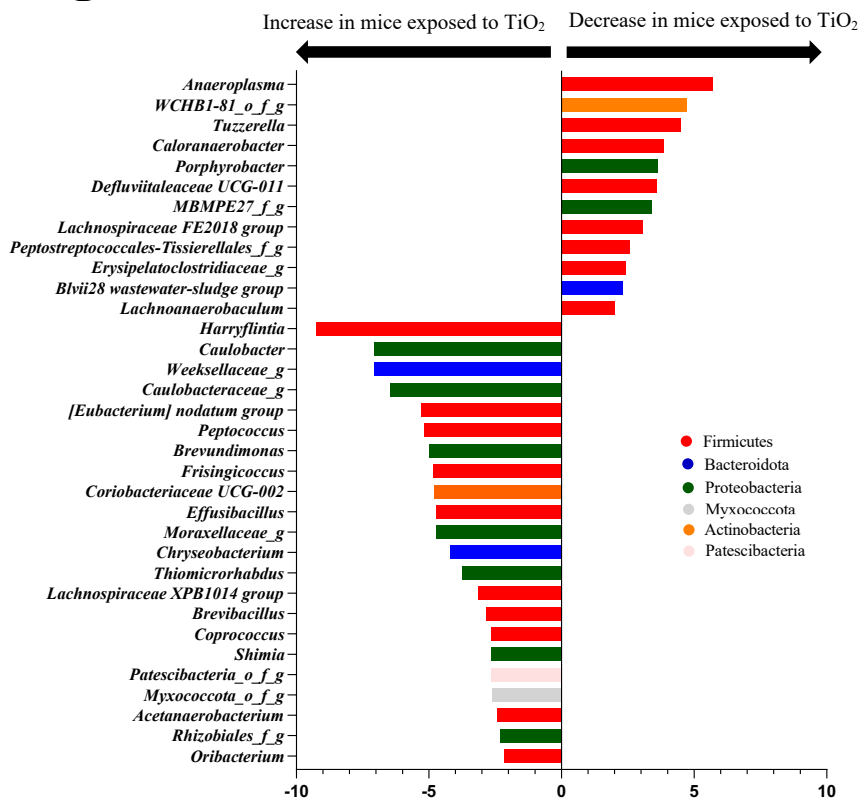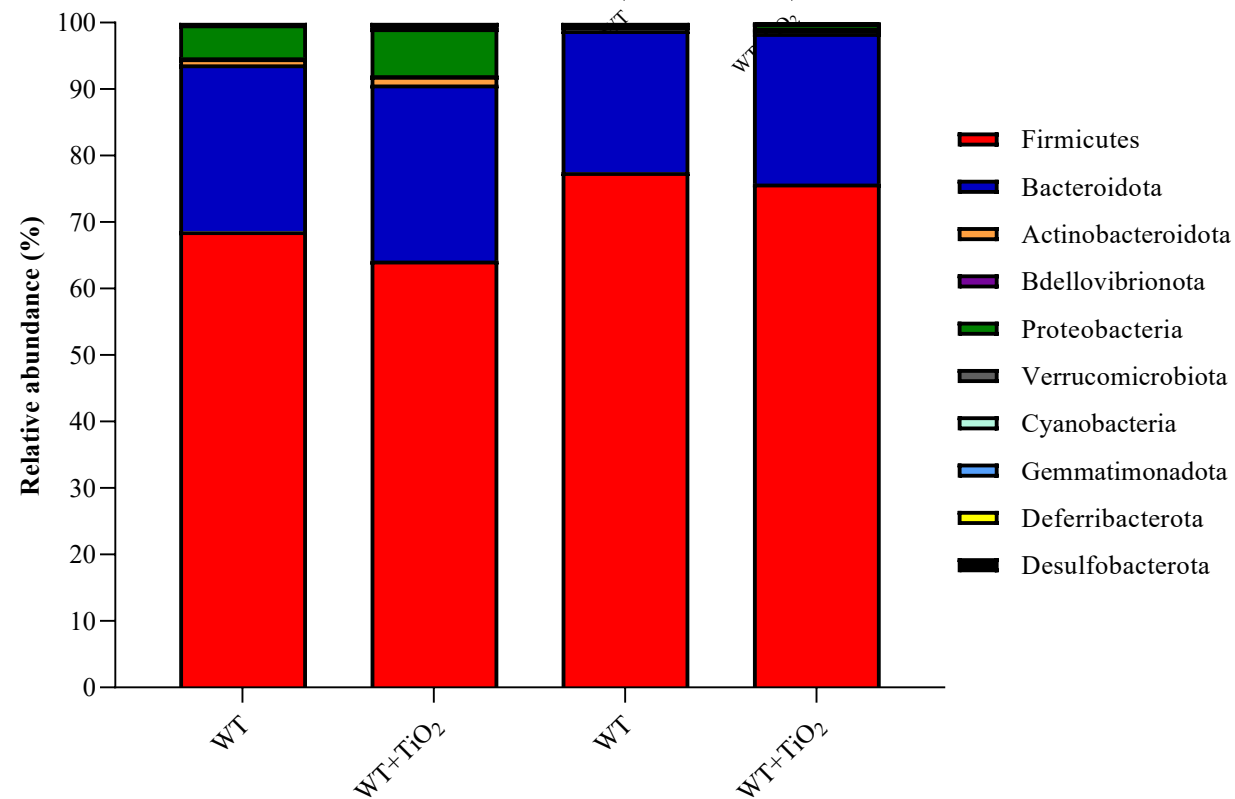

Supplement: Supplementary file 3 — Additional file 3. Fig. S3: Impact of perinatal exposure to foodborne TiO2 on colonic microbiota at day 50. (A-E) Wild type female mice have been exposed to TiO2 (9 mg/Kg of BW/Day) during the perinatal period including gestational and lactating periods. Weaning pups were also exposed to TiO2 (9 mg/Kg of BW/Day) until day 50 after birth (A). Then at day 50 after birth, pups have been sacrificed and the structure of the colonic mucosa-associated microbiota has been monitored by 16S rRNA gene sequencing (B-D). (B) Alpha diversity of colonic mucosal microbiota from exposed or non-exposed mice to foodborne TiO2 at day 50 after birth. (C-D) Composition of colonic microbiota at phyla level (C) and Fold changes 2 for bacterial genera significantly perturbed (D) from exposed or non-exposed mice to foodborne TiO2 at day and 50 after birth. Data are expressed as median ± SEM and were analysed by Mann and Whitney test. At least n = 8 per group. [file 12989_2023_555_MOESM3_ESM.pdf]

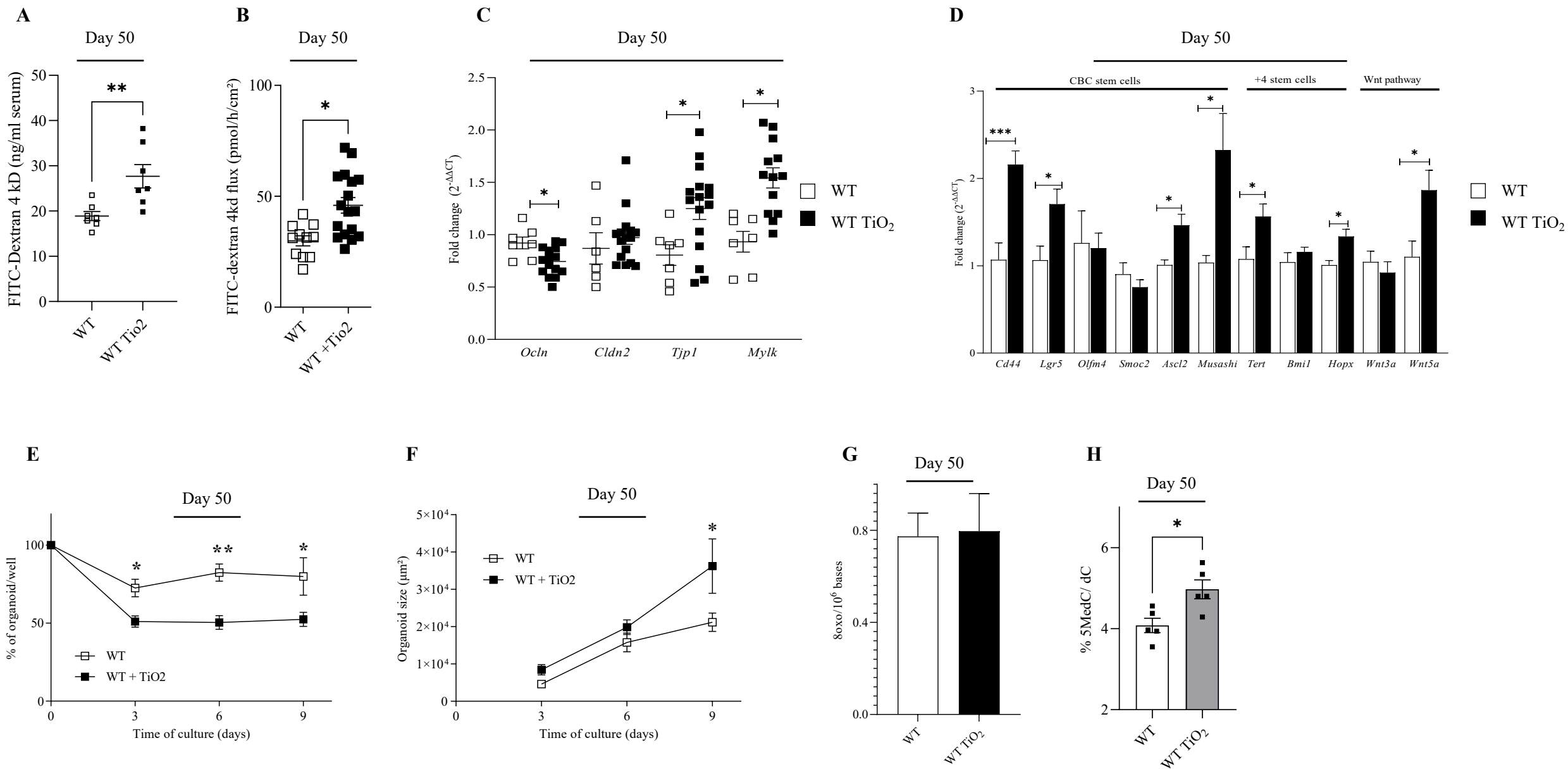

Supplement: Supplementary file 4 — Additional file 4. Fig. S4: Impact of perinatal exposure to foodborne TiO2 on colonic epithelium at day 50. (A-D) Wild type female mice have been exposed to TiO2 (9 mg/Kg of BW/Day) during the perinatal period including gestational and lactating periods. Weaning pups were also exposed to TiO2 (9 mg/Kg of BW/Day) until day 50 after birth (A-D). Then at day 50 after birth, pups have been sacrificed and several parameters including permeability (A and B), mRNA expression (C and D), abilities of intestinal stem cells to survive (E) and proliferate (F), oxidative stress (G) and DNA methylation (H) were assessed. (A) In vivo permeability was determined by measuring the level of plasmatic FITC-dextran 4 kDa, 3 h following oral administration. (B) Colonic permeability was monitored by measuring the flux of FITC-dextran 4 kDa across colonic biopsies mounted in Ussing chamber for 1 h. (C) mRNA expression of Occludin (Ocl), Claudin 2 (Cldn 2) Tight junction protein 1 (Tpj1) and myosin light chain kinase (Mlck) was studied at day 50 after birth. (D) Colonic mRNA expression of CD44, Leucine-rich repeat-containing G-protein coupled receptor 5 (Lgr5), Olfactome-din 4 (Olfm4), SPARC-related modular calcium-binding protein 2 (Smoc2), Achaete-scute complex homolog 2 (Ascl2), Musashi RNA-binding protein 1 (Musashi), Telomerase reverse transcriptase (Tert) and B lymphoma Mo-MLV insertion region 1 homolog (Bmi1), homeodomain-only protein homeobox (Hopx), canonical (Wnt3a) and non-canonical (Wnt5a). (E) The organoid survival has been monitored by measuring the percentage of viable organoids according to the time culture. (F) The organoid growth has been studied by measuring the organoid surface according to the time culture. (G) Oxidative stress has been monitored into the epithelial cells from the colonic based crypt of mice perinatally exposed or not to TiO2. (H) The quantity of cytosine methylated of DNA from the epithelial cells from the colonic based crypt of mice perinatally expose [file 12989_2023_555_MOESM4_ESM.pdf]

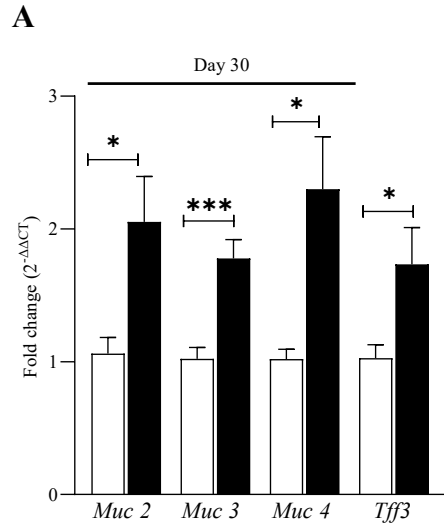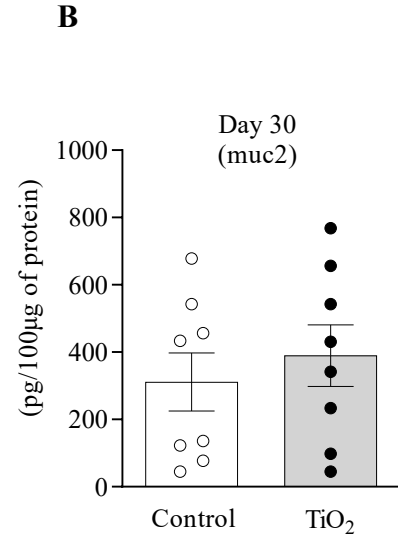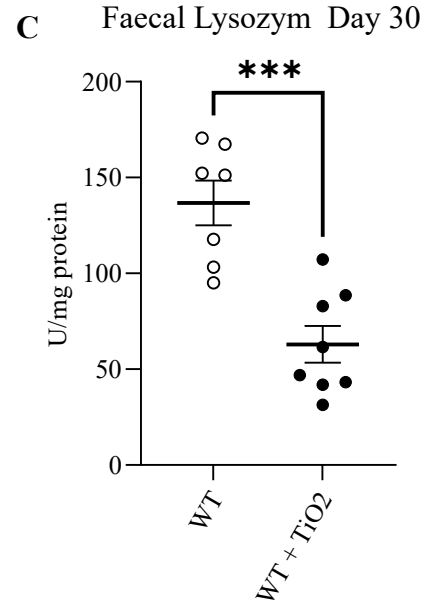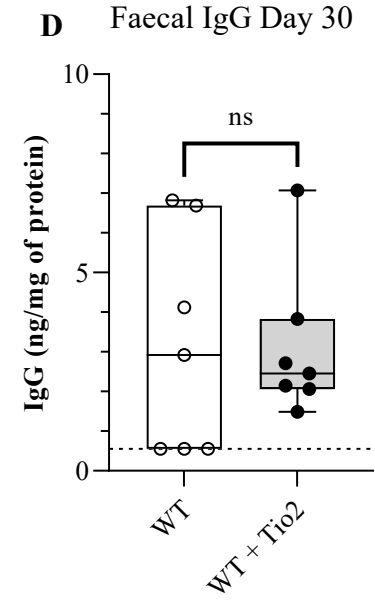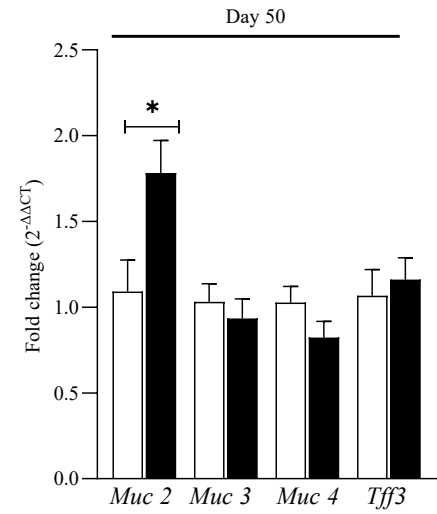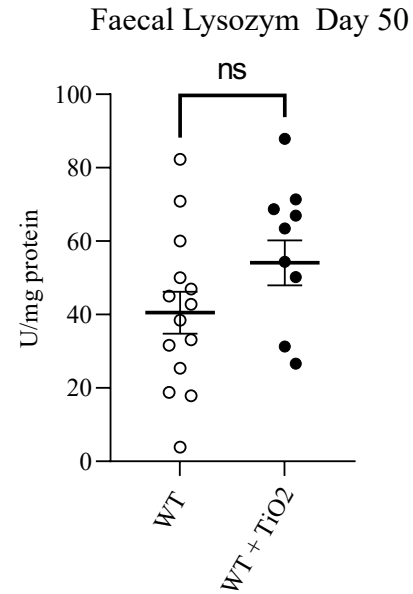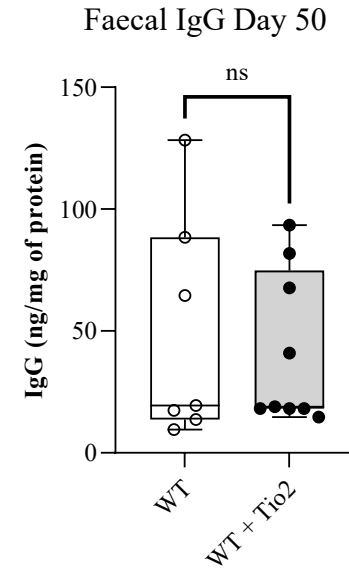

Supplement: Supplementary file 5 — Additional file 5. Fig. S5: Impact of perinatal exposure to foodborne TiO2 on mucus, antimicrobial peptides and immunoglobulins. (A-D) Wild type female mice have been exposed to TiO2 (9 mg/Kg of BW/Day) during the perinatal period including gestational and lactating periods. Then, at days 30 or 50 after birth, pups have been sacrificed and several parameters including colonic mRNA expression of mucin 2 (Muc2), mucin 3 (Muc3), mucin 4 (Muc4) and Trefoiled factor 3 (Tff3) (A, B), faecal levels of lysozym (C) and IgG (D). Data are expressed as mean ± SEM and were analysed by Student’s t-test. *p < 0.05 and ***p < 0.001 vs. control group. At least n = 5 per group. [file 12989_2023_555_MOESM5_ESM.pdf]

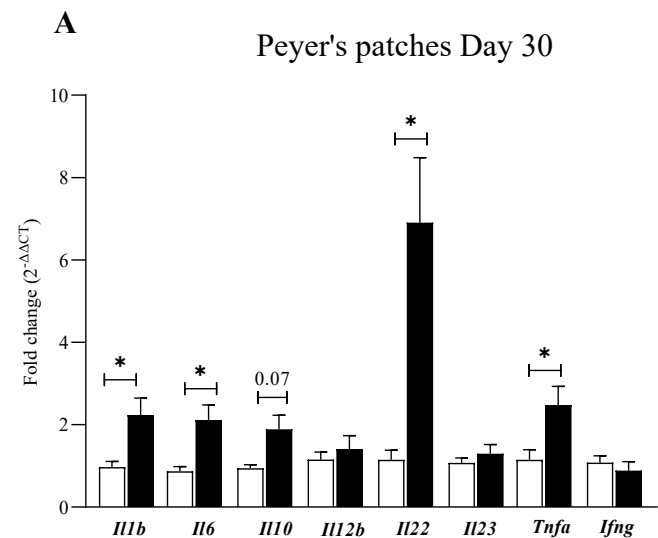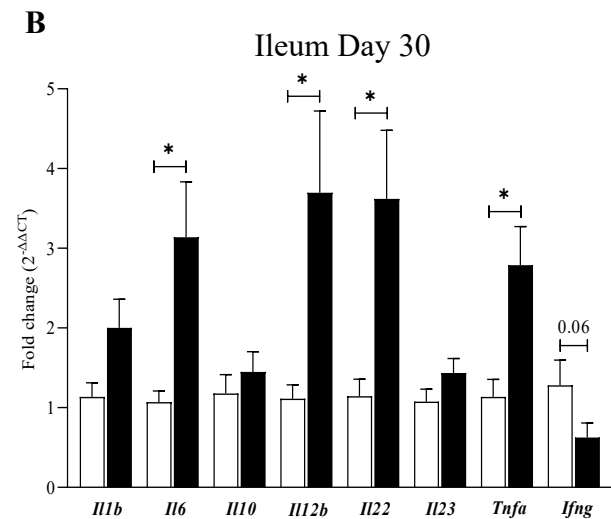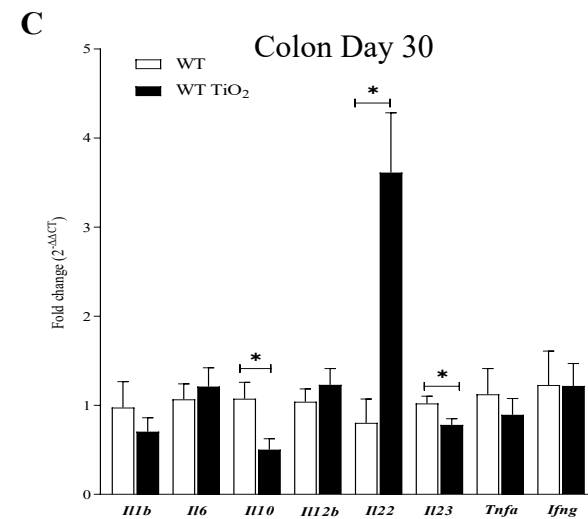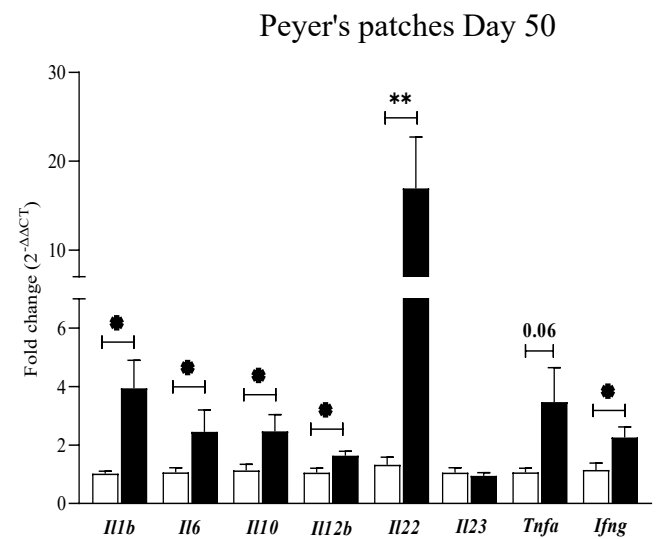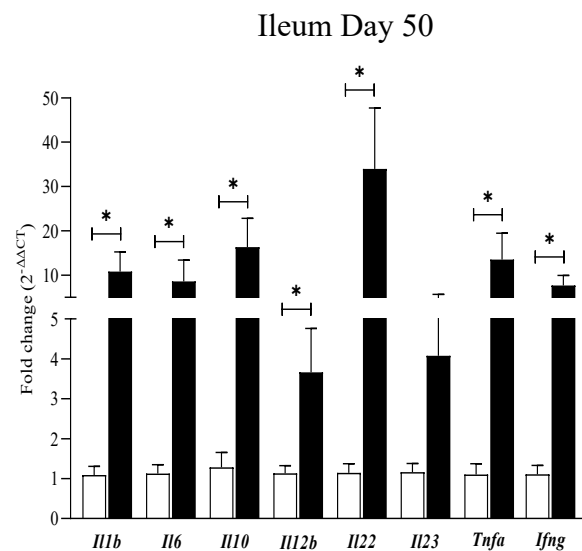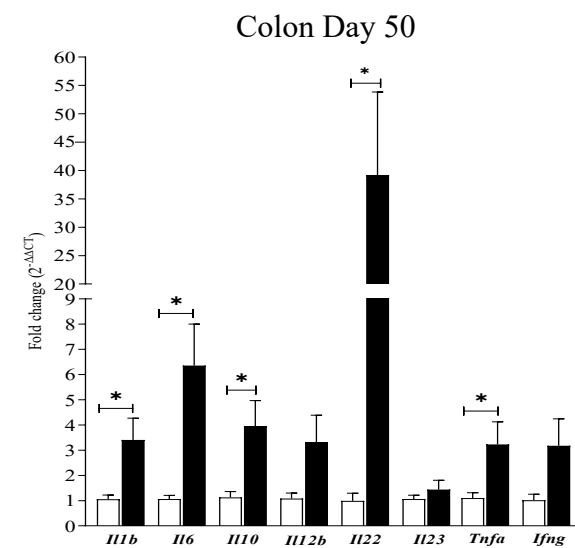

Supplement: Supplementary file 6 — Additional file 6. Fig. S6: Impact of perinatal exposure to TiO2 foodborne on intestinal immune system. (A-C) Wild type female mice have been exposed to TiO2 (9 mg/Kg of BW/Day) during the perinatal period including gestational and lactating periods. Weaning pups were also exposed to TiO2 (9 mg/Kg of BW/Day) until day 50 after birth. Then, at days 30 or 50 after birth, pups have been sacrificed and several parameters including mRNA expression have been monitored. (A–C) On Peyer’s patches (A) and scrapped ileal (B) and colon (C)), mRNA expression of Il1b, Il6, Il10, Il12, Il22, Il23, TNFa, IFNg, was monitored at days 30 and 50 after birth. Data are expressed as mean ± SEM and were analysed by Student’s t-test. *p < 0.05 and **p < 0.01 vs. control group. At least n = 8 per group. [file 12989_2023_555_MOESM6_ESM.pdf]

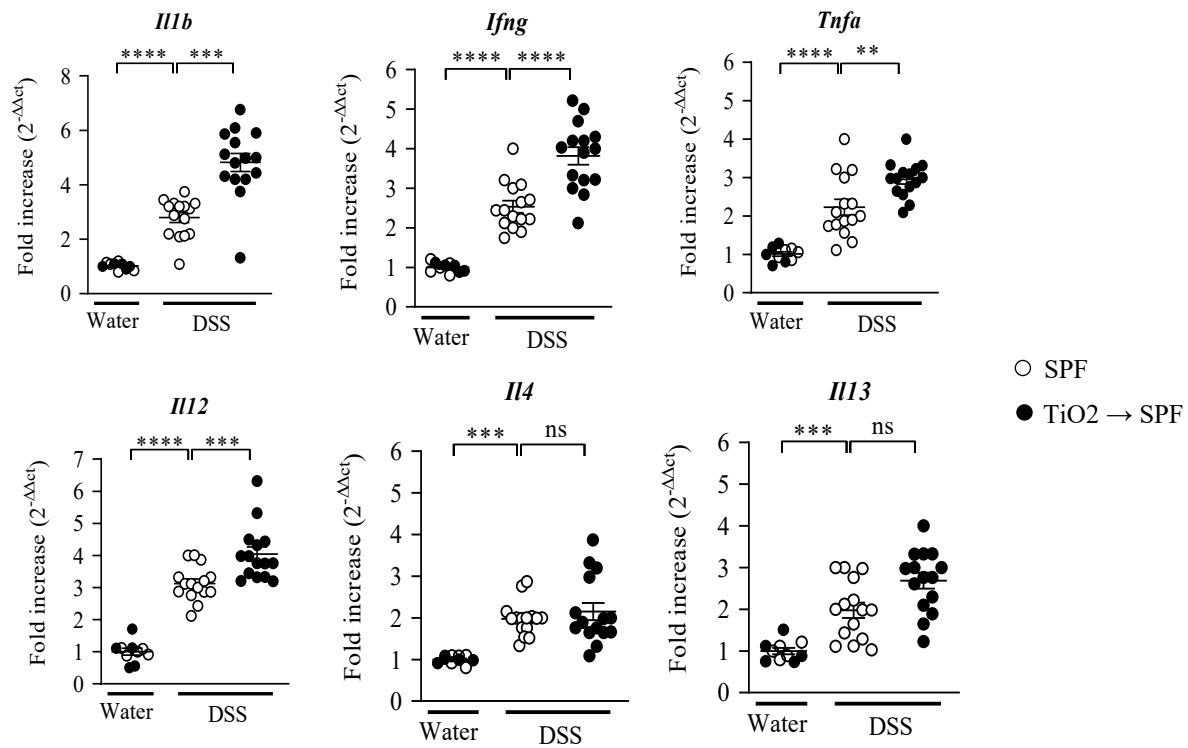

Supplement: Supplementary file 7 — Additional file 7. Fig. S7: Impact of perinatal exposure to foodborne TiO2 on cytokines mRNA expression in context of colitis. Wild type female mice have been exposed to TiO2 (9 mg/Kg of BW/Day) during the perinatal period including gestational and lactating periods. At 14 weeks of age, colitis has been orally induced by introducing Dextran Sulfate Sodium (DSS) into drinking water at 2% for 7 days followed by 7 days of regular water then 7 days of DSS. Then, at the end of the DSS procedure, mice have been sacrificed and cytokine mRNA expression have been monitored. Data are expressed as mean ± SEM and were analysed by Student’s t-test. **p < 0.01; ***p < 0.001 and ****p < 0.0001 vs. control group. At least n = 5 per group. [file 12989_2023_555_MOESM7_ESM.pdf]

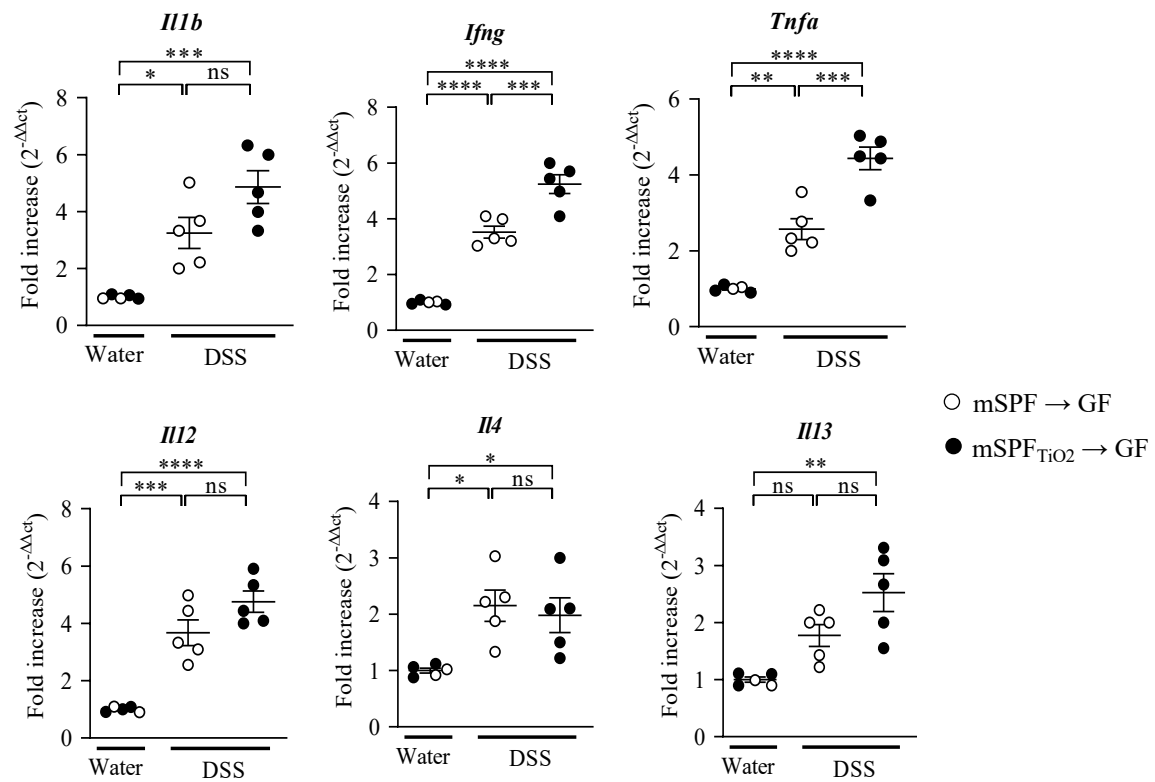

Supplement: Supplementary file 8 — Additional file 8. Fig. S8: Impact of gut microbiota dysbiosis induced by perinatal exposure to TiO2 on the cytokines mRNA expression in context of colitis. Germ free mice female have been exposed to gut microbiota dysbiosis induced by perinatal foodborne until the weaning i.e. postnatal day 30. At 14 weeks of age, colitis has been orally induced by introducing Dextran Sulfate Sodium (DSS) into drinking water at 2% for 7 days followed by 7 days of regular water then 7 days of DSS. Then, at the end of the DSS procedure, mice have been sacrificed and cytokines mRNA expression have been monitored. Data are expressed as mean ± SEM and were analysed by Student’s t-test. *p < 0.05; **p < 0.01; ***p < 0.001 and ****p < 0.0001 vs. control group. At least n = 5 per group. [file 12989_2023_555_MOESM8_ESM.pdf]

A

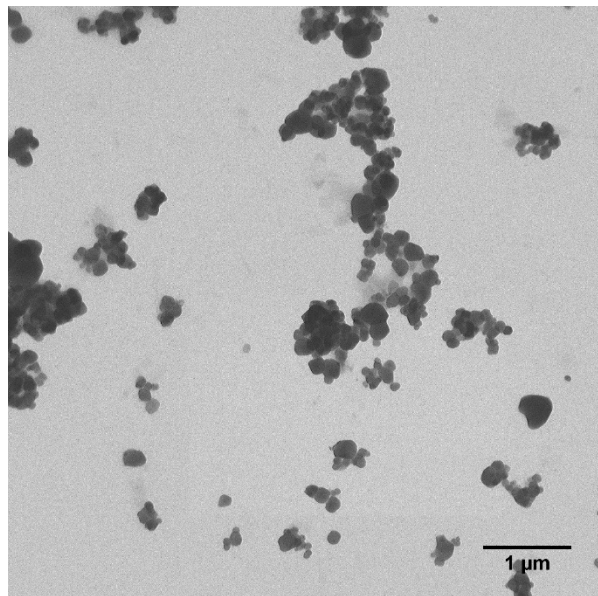

B

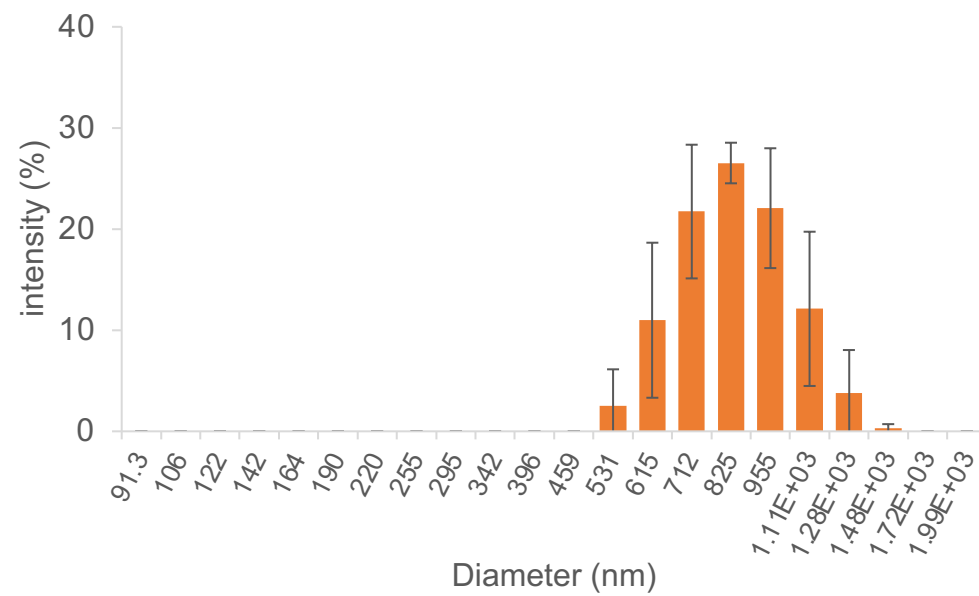

Supplement: Supplementary file 9 — Additional file 9. Fig. S9: Physico-chemical characteristics of TiO2 particles. (A) Transmission electron microscopy images of TiO2 particles, recorded on a JEOL 1200EX TEM operating at 80 kV (Grenoble Institut des Neurosciences, Grenoble, France). (B) Size distribution of TiO2 particles in the drinking water, measured via dynamic light scattering on a Malvern nanoZS zetasizer. [file 12989_2023_555_MOESM9_ESM.pdf]

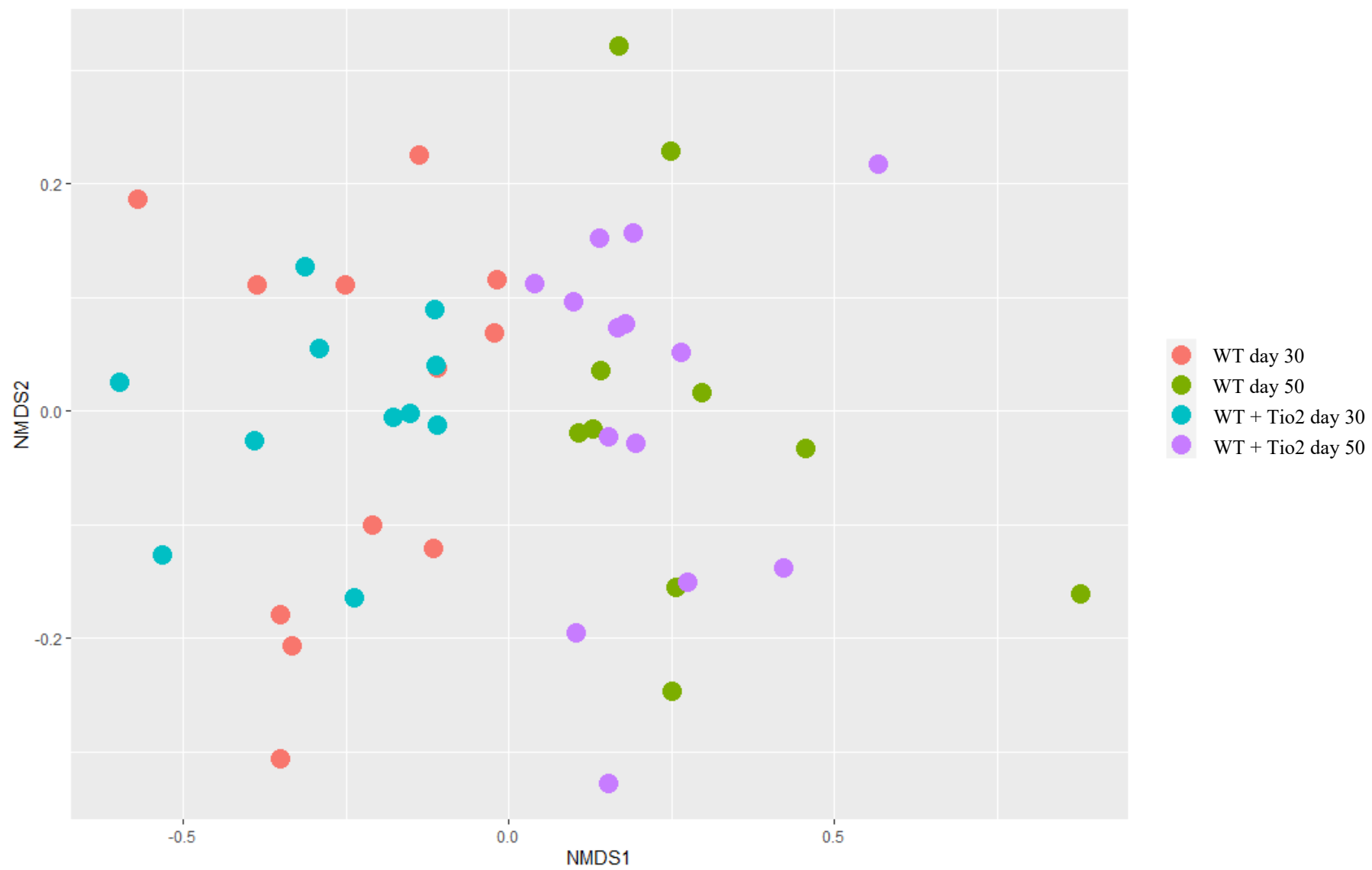

Supplement: Supplementary file 10 — Additional file 10. Fig. S10: Beta diversity analysis of colonic microbiota. Beta diversity of the colonic bacterial community (all bacterial taxa considered) was analyzed using multidimensional non-metric scaling (NMDS) plots generated by Bray–Curtis dissimilarity index. The four mouse groups are represented by different colors: pink for mice non-exposed at TiO2 at days 30, green for mice non-exposed at TiO2 at days 50, blue mice exposed at TiO2 at days 30 and purple for exposed at TiO2 at days 50. [file 12989_2023_555_MOESM10_ESM.pdf]
