# Supplementary material for: Perinatal foodborne titanium dioxide exposure-mediated dysbiosis predisposes mice to develop colitis through life
Source: Part Fibre Toxicol. 2023 Nov 23;20:45. doi: 10.1186/s12989-023-00555-5 (PMC10666382; doi:10.1186/s12989-023-00555-5)
Supplement: Supplementary file 11 — Additional file 11. Table S1: Sequence of oligonucleotides used for RT-qPCR experiments. [file 12989_2023_555_MOESM11_ESM.pdf]

**Genes**	**Forward 5'-3'**	**Reverse 3'- 5'**
Mucin 2 (*Muc2*)	CTGTTTTCCCCGTCTGTGGTT	CTCTGCCTGCTGGCTTTCAT
Mucin 3 (*Muc3*)	CTGTAGTGTGGGGTGCTGAC	TGACTGCCGAGACTCCTACA
Mucin 4 *(Muc4*)	CCCTCACTACATGGGGACAC	TGGCTACAAAGGCTACCACC
Trefoiled Factor 3 (*Tff3*)	GGAGCCTGGACAGCTTCAAA	ACGTTGGCCTGTCTCCAAG
Tight junction protein 1 (*Tjp1*)	ACCCGAAACTGATGCTGTGGATAG	AAATGGCCGGGCAGAACTTGTGTA
Tight junction protein 2 (*Tjp2*)	TGCAATTCCAAATCCAAACC	GTGATTTTCTTCAACCCGGA
Occludin (*Ocl*)	TTCTCCCGCAACTGGCATC	GAGTGAAGAGTACATGGCTGCT
Claudin 2 (C*ldn2*)	AGGCATCTAGAAAACGGAGCC	GGACCTAGTCCTTGTCCCCA
Myosin Light Chain Kinase (*Mylk*)	CTCACTGGTCTGAGCATCGTC	AATTCATCGTCCTCGCCCAA
CD44	GGAGTACTTGCAACAGGCATCT	ACGAGTGCAACTACAGCCTT
Leucine-rich repeat-containing G-protein coupled receptor 5 (*Lgr5*)	CAAGGTCCCGCTCATCTTGA	CAGTGTTGTGCATTTGGGGG
Olfactomedin-4 (OLFM4)	CAGCTGCCTGGTTGCCTCCG	GGCAGGTCCCATGGCTGTCC
SPARC-related modular calcium-binding protein 2 (*Smoc2*)	GCAGGGAAAGCAGATGATGC	CTCGGTCCAGAGTGTAGGGT
Achaete-scute family bHLH transcription factor 2 (*Ascl2*)	GCCTACTCGTCGGAGGAA	CCAACTGGAAAAGTCAAGCA
Musashi RNA-binding protein 1 (*Musashi*)	CGAGCTCGACTCCAAAACAAT	GGCTTTCTTGCATTCCACCA
Telomerase reverse transcriptase (*Tert*)	GGAGTACTTGCAACAGGCATCT	ACGAGTGCAACTACAGCCTT
B lymphoma Mo-MLV insertion region 1 homolog (*Bm1*)	TCCCCACTTAATGTGTGTCCT	CTTGCTGGTCTCCAAGTAACG
Homeodomain-only protein X (*Hopx*)	TCTCCATCCTTAGTCAGACGC	GGGTGCTTGTTGACCTTGTT
Wnt3A	AATGTCCTCACTACAGCCGC	TGTTCTGGACAAAGCCACCC
Wnt5A	GGGTTATTCATACCTAGAGACCACC	GCTGCAACAGACTGGAGAGG
Il1b	ACCTTCCAGGATGAGGACATGAG	CATCCCATGAGTCACAGAGGATG
Il6	CTCTGCAAGAGACTTCCATCCAGT	CGTGGTTGTCACCAGCATCA
Il10	AGGCGCTGTCATCGATTTCTC	TGCTCCACTGCCTTGCTCTTA
Il12b	GAGTTCTCTGCCTCTCTCGC	ATGAGGAGCTGGCTTTGGTC
Il22	AGGTGGTGCCTTTCCTGACC	ACCGCTGATGTGACAGGAGC
Il23	TGCAGCTTTGTCACAGGTCA	CTGTACCTGCCTGCTCTGTC
Tnfa	AATGGCCTCCCTCTCATCAG	GCTACGACGTGGGCTACAGG
Ifng	CAGCAACAGCAAGGCGAAA	AGCTCATTGAATGCTTGGCG
Gapdh	TGTAGACCATGTAGTTGAGGTCA	AGGTCGGTGTGAACGGATTTG
